# EEG Microstate-Specific Functional Connectivity and Stroke-Related Alterations in Brain Dynamics

**DOI:** 10.3389/fnins.2022.848737

**Published:** 2022-05-11

**Authors:** Zexuan Hao, Xiaoxue Zhai, Dandan Cheng, Yu Pan, Weibei Dou

**Affiliations:** ^1^Department of Electronic Engineering, Beijing National Research Center for Information Science and Technology (BNRist), Tsinghua University, Beijing, China; ^2^Department of Rehabilitation Medicine, School of Clinical Medicine, Beijing Tsinghua Changgung Hospital, Tsinghua University, Beijing, China

**Keywords:** EEG, microstates, brain dynamics, functional connectivity, stroke, machine learning, lower extremity motor function

## Abstract

The brain, as a complex dynamically distributed information processing system, involves the coordination of large-scale brain networks such as neural synchronization and fast brain state transitions, even at rest. However, the neural mechanisms underlying brain states and the impact of dysfunction following brain injury on brain dynamics remain poorly understood. To this end, we proposed a microstate-based method to explore the functional connectivity pattern associated with each microstate class. We capitalized on microstate features from eyes-closed resting-state EEG data to investigate whether microstate dynamics differ between subacute stroke patients (*N* = 31) and healthy populations (*N* = 23) and further examined the correlations between microstate features and behaviors. An important finding in this study was that each microstate class was associated with a distinct functional connectivity pattern, and it was highly consistent across different groups (including an independent dataset). Although the connectivity patterns were diminished in stroke patients, the skeleton of the patterns was retained to some extent. Nevertheless, stroke patients showed significant differences in most parameters of microstates A, B, and C compared to healthy controls. Notably, microstate C exhibited an opposite pattern of differences to microstates A and B. On the other hand, there were no significant differences in all microstate parameters for patients with left-sided vs. right-sided stroke, as well as patients before vs. after lower limb training. Moreover, support vector machine (SVM) models were developed using only microstate features and achieved moderate discrimination between patients and controls. Furthermore, significant negative correlations were observed between the microstate-wise functional connectivity and lower limb motor scores. Overall, these results suggest that the changes in microstate dynamics for stroke patients appear to be state-selective, compensatory, and related to brain dysfunction after stroke and subsequent functional reconfiguration. These findings offer new insights into understanding the neural mechanisms of microstates, uncovering stroke-related alterations in brain dynamics, and exploring new treatments for stroke patients.

## Introduction

It is now a consensus that the brain at rest is not truly “at rest”, and the spontaneous brain activity exhibits complex dynamic spatiotemporal configurations (Raichle et al., 2001; Fox and Greicius, 2010; Pirondini et al., 2017). Of note, spontaneous brain activity can predict behavioral performance, and intrinsic activity plays a basic and functional role in brain function (Spisak et al., 2020). On the other hand, brain injury (e.g., stroke) causes behavioral deficits as well as widespread structural and functional network dysfunction (Salvalaggio et al., 2020). Motor and sensory impairments are the two most common deficits after stroke, affecting approximately 85 and 50% of stroke patients, respectively (Wu et al., 2015; Ramsey et al., 2017). To date, using neurophysiological techniques, a tremendous number of studies based on spectral analysis, functional connectivity, and graph theory analysis have been reported in the field of brain injury mechanisms and stroke rehabilitation (Wu et al., 2015; Stinear, 2017; Trujillo et al., 2017; Mane et al., 2019; Chiarelli et al., 2020; Ros et al., 2022). These studies laid an important foundation for our understanding of stroke-related neurological alterations and neuroplasticity and for finding neural markers that can indicate prognosis and outcomes. However, given the intrinsic non-stationary nature of brain neural signals, static features are not time-resolved and lose the information about the time dimension. Dynamic analysis methods may better reflect the full profile of brain activity and capture critical features of the spatiotemporal dimensions in health and disease (Kabbara et al., 2017; Chang et al., 2018; O’Neill et al., 2018; Bonkhoff et al., 2020; Wang et al., 2020). Dynamic functional connectivity is the most commonly used method in functional magnetic resonance imaging (fMRI) studies to investigate the connectivity dynamics in stroke patients. It has shown that stroke alters the brain’s preference for distinct connectivity states (Bonkhoff et al., 2020; Wang et al., 2020). However, the sliding-window approach, which is typically utilized in dynamic functional connectivity, has the limitation of requiring a *prior* unknown window width (O’Neill et al., 2018). Windows that are too short or too long will not capture the true temporal dynamics of the brain. Several alternative approaches have been applied to electrophysiological data to describe the brain dynamics, including hidden Markov models (HMMs) (Vidaurre et al., 2018; Bai et al., 2021a) and microstate analysis (Pascual-Marqui et al., 1995; van de Ville et al., 2010; Zanesco et al., 2020). Spatially distinct patterns of oscillatory power and coherence at the source level have been observed in health and disorders of consciousness by HMMs (Vidaurre et al., 2018; Bai et al., 2021a). Nonetheless, it remains unclear whether the assumptions underlying HMMs are met in resting-state EEG (Gschwind et al., 2015; von Wegner et al., 2016). In addition, little is known about the electromagnetic properties of the brain injury regions, and techniques for constructing accurate head models of patients with brain injuries (e.g., stroke) have not yet been well developed. Microstates reflect short periods (∼100 ms) of quasi-stable brain states that evolve in time, resulting from the synchronous and coordinated activity of brain networks (Pascual-Marqui et al., 1995; Koenig et al., 2002; Zanesco et al., 2020). Importantly, microstate analysis is traditionally at the sensor level and needs minimal assumptions regarding the properties of the neural signals.

Numerous studies based on EEG have demonstrated that resting-state functional connectivity and microstate analyses are successful and valuable methods for studying neurological and psychiatric diseases (Khanna et al., 2015; Zappasodi et al., 2017; Zuchowicz et al., 2018; Eldeeb et al., 2019; Musaeus et al., 2019a; Riahi et al., 2020; Tait et al., 2020). Additionally, there are associations between EEG microstates and fMRI resting-state networks (RSNs) (Britz et al., 2010; van de Ville et al., 2010; Custo et al., 2017). Furthermore, motor scores are related to EEG features (e.g., functional connectivity) of spontaneous brain activity (Riahi et al., 2020; Hoshino et al., 2021). Given the tight link between microstates and brain networks of spontaneous brain activity, we speculated that microstate dynamics could reflect the motor capacity (e.g., lower/upper limb function) to some extent (Pirondini et al., 2017; Spisak et al., 2020; Zhang et al., 2021). However, the current microstate studies have mainly focused on the cognitive functions of the brain in health (Brechet et al., 2019; Pirondini et al., 2020; Zanesco et al., 2020) and diseases like schizophrenia (da Cruz et al., 2020) and Alzheimer (Musaeus et al., 2019b; Tait et al., 2020). Only a limited number of studies focused on patients with brain injuries, such as stroke (Zappasodi et al., 2017) and consciousness of disorders (Gui et al., 2020; Bai et al., 2021b). To date, only one study (to our knowledge) with the complete microstate analysis (Zappasodi et al., 2017) focused on stroke (at the acute stage). This research suggested that microstate B duration explained the 11% of the effective recovery of the National Institute of Health Stroke Scale (NIHSS), and there was a significant difference between patients with left-sided and right-sided stroke in parameters of microstate C and D. Further in-depth research is needed to verify and explore the stroke-related alterations in microstate dynamics.

There are still three critical questions that need to be deeply investigated. First, is each microstate class associated with a specific functional connectivity pattern? Several studies have explored state-wise functional connectivity in different scenarios (Comsa et al., 2019; Duc and Lee, 2019), but the connectivity pattern corresponding to each microstate class in the resting state is still unclear. In addition, according to previous studies, each microstate template has distinct topographic distribution and bright characteristics (Michel and Koenig, 2018; Li et al., 2021; Zanesco et al., 2021). Therefore, we assumed that each microstate class corresponds to a unique pattern of functional connectivity. It is instructive for addressing this question to extend our understanding of the mechanisms underlying microstates and help us explain microstate dynamics. Second, how do the microstate parameters evolve in time after stroke, and how are they perturbed or altered by stroke? Third, do microstate parameters encode information that reflects the motor capacity of the lower limbs? Lower limb motor function is an important basis for restoring walking function and activities of daily living after stroke. However, currently few studies have assessed lower limb function by neural features derived from EEG (Sebastian-Romagosa et al., 2020; Hoshino et al., 2021). The answers to the latter two questions will help us to explore the crucial characteristics of stroke patients, the possible patterns of brain functional reorganization, and the possibility of using microstate dynamics for functional assessment.

In the current study, we proposed a microstate-based approach and leveraged the EEG datasets of patients at two-time points (i.e., before and after the rehabilitation therapy) and healthy controls to explore the three aforementioned questions. The Lower-extremity part of Fugl-Meyer Assessment (FMAL) was used to evaluate the lower limb functional status of stroke patients at the two-time points (Fugl-Meyer et al., 1975). Previous research has suggested that intra-cortical alpha oscillations primarily account for the emergence of microstate classes (Milz et al., 2017), and most microstate studies are based on larger bandwidths such as 2–20 or 1–40 Hz (Khanna et al., 2015). Here, we used the EEG signal in the 1–20 Hz frequency band for microstate analysis. Phase-locked synchronization of neural signals in the brain may be the key mechanism for brain information integration (Michel and Koenig, 2018). Therefore, in this study, functional connectivity in the alpha band was constructed by the phase-locking value (PLV) method (Lachaux et al., 1999; Duc and Lee, 2019; Yao et al., 2021). Microstate-specific connectivity was computed by connected instantaneous phase signals belonging to a particular microstate class. We compared one microstate-specific functional connectivity with the others to examine whether there were differences among microstate-wise connectivity. The results were also validated in an independent EEG dataset (Babayan et al., 2019). In addition, due to the functional and structural deficits after stroke, we predicted that some microstate parameters would be significantly different in stroke patients compared to those in healthy controls. We also tested how microstate features changed after rehabilitation training, and whether there were significant differences in microstate features between patients with left-sided and right-sided stroke. Moreover, we utilized support vector machine (SVM) models to investigate their ability to distinguish stroke patients from healthy controls by only microstate features. Finally, we explored the cross-sectional correlations between the microstate features and FMAL scores. This may allow us to identify potential neural markers that provide insight into lower limb function recovery after stroke.

In sum, we explored the functional connectivity patterns underlying microstates and investigated how microstate dynamics are altered in stroke patients at two-time points compared to healthy individuals and the associations between microstate features and FMAL scores. No such study exists for microstates.

## Materials and Methods

### Participants

#### Patients

Fifty-nine patients were recruited, and 31 patients (mean age 56.7 years with range 31.7–77.4; *SD* = 12.1; 29 right-handed; 24 males; 19 with left-sided stroke) satisfied the post-enrollment inclusion criteria. No statistical methods were used to predetermine the sample sizes in this study, but our sample sizes were similar to those reported in previous research (Pirondini et al., 2020; Wang et al., 2021).

#### Inclusion Criteria of Patients

(1) Aged 30–80. (2) First-ever unilateral brain lesion. (3) Subacute stroke (2 weeks to 6 months poststroke). (4) Sufficient cognition (Mini-Mental State Examination, MMSE score > 21). (5) Moderate-to-severe paralysis (Brunnstrom score ≤ IV). (6) No other diagnosis substantially affecting the lower limbs. (7) No other neurological or psychiatric disorders. (8) Medically stable.

#### Healthy Controls

Twenty-three healthy controls (mean age 58.9 years with range 31.5–73.2; *SD* = 12.0; 21 right-handed; 13 males) were recruited for this study. In addition, an external site EEG dataset of healthy subjects (*N* = 32; age range 30–80; 29 right-handed; 21 males) selected from the “Mind-Brian-Body dataset” (Babayan et al., 2019) was used to validate the stability of the results of microstate-specific functional connectivity (Supplementary Table 1). To distinguish the external site EEG dataset from the healthy controls in this study, “LEMON” was used to refer to the external site dataset.

The study was conducted according to the tenets of the Declaration of Helsinki, the guidelines for Good Clinical Practice, and the Consolidated Standards of Reporting Trials (CONSORT), approved by the Ethics Committee of Beijing Tsinghua Changgung Hospital (18172-0-01). All subjects provided written informed consent before participating in the study.

### Treatment Protocol and Clinical Evaluation

All patients received 10-session ankle stretching training using the robot-aided stretching (five times a week over 2 weeks, 20 min/session). An ankle rehabilitation robot (Beijing LTK Science and Technology Co., Ltd., Beijing, China) was used for intervention. The ankle rehabilitation robot was driven by a servomotor controlled by a digital signal processor. The stretching protocol has been described in detail in our previous research (Zhai et al., 2021). During the 2 weeks, all patients received standard medical care and rehabilitation, which consisted of routine physiotherapy (PT) and occupational therapy (OT). Patients completed a 1-h PT session and 1-h OT per day, 5 days per week, for a total of 10 sessions. PT included continued movement exercises for hemiplegia, muscle strength training, and balance and walking function exercises. OT focused on rehabilitation of arm and hand movements used in daily activities. Subjects were evaluated before and after the interventions by a designated physiotherapist. FMAL (0–34 points) was used to evaluate the lower limb motor function of stroke patients and was conducted at two-time points: before the therapy (T0, baseline) and immediately after 10 training sessions (T1) (Fugl-Meyer et al., 1975; Gladstone et al., 2002; See et al., 2013). In addition, EEG data were also collected for each patient at two-time points (Supplementary Figure 1A). Group characteristics and clinical information are presented in Table 1.

**TABLE 1:** Sample demographics and clinical information.

	Patients (*N* = 31)	Controls (*N* = 23)	*p*	Effect size
Age (M ± *SD*, range)	56.7 ± 12.1, 31.7–77.4	58.9 ± 12.0, 31.5–73.2	0.52	−0.18
Gender (female/male)	7/24	10/13	0.10	0.22
Handedness (left/right)	2/29	2/21	0.76	0.04
Lesion side (left/right)	19/12			
Stroke type (hemorrhagic/ischemic)	6/25			
Days poststroke at T0 (M ± *SD*, range)	65.9 ± 39.7, 15–154			

*M ± SD: mean ± standard deviation. The two-sample t-test for age and Pearson’s chi-squared tests for gender and handedness were performed between patients and controls. The effect size was estimated using Cohen’s d for age and Cramer’s V for gender and handedness.*

### EEG Data Acquisition and Preprocessing

Eyes-closed resting-state EEG data were acquired from a 64-channel electrode cap (NeuSen W64, Neuracle, China), with 59 scalp electrodes (Ag/AgCl) placed according to the 10-10 international system. The data file of the channel locations is provided in Supplementary Material. EEG data were recorded using a Neuracle amplifier with 24-bit resolution and a sampling rate of 1,000 Hz, and lowpass filtered with the cutoff frequency (−3 dB) at 250 Hz for between approximately 3–5 min. The reference electrode was located at CPz, and the ground electrode was located at AFz. During the recording, the impedance was kept below 20 kΩ for all scalp electrodes. EEG data were recorded in a specific, dimly lit, sound-attenuated, but not electrically shielded room. An investigator focused on the status of the participants, and they were requested to minimize their movements and remain awake.

EEG data were preprocessed offline in MATLAB (R2020b, Mathworks, Natick, MA, United States) using the EEGLAB toolbox (version 2019.0, Swartz Center for Computational Neuroscience, San Diego, CA, United States) and its extensions in combination with some custom MATLAB scripts. All preprocessing steps consistently maintained double-precision computations. FIR filters were used in this study and designed using the function *pop_firws()* with a Hamming window. First, EEG data were lowpass filtered (order: 660, transition width: 5.0 Hz, −6 dB cutoff frequency: 45 Hz) to remove the line noise and then resampled to 250 Hz. In addition, low-frequency noise was attenuated using a high-pass filter (order: 826, transition width: 1 Hz, −6 dB cutoff frequency: 0.5 Hz). Then, bad channels and EEG segments containing large, non-stereotype artifacts were discarded semi-automatically. The signal of the bad channels was interpolated through spherical interpolation and then EEG data were re-referenced to the common average and the signal of the original reference CPz channel was restored. Next, EEG data were decomposed by independent component analysis (ICA) using the default *binica* method in the EEGLAB toolbox. Artifactual components (e.g., eye blinks, movement, channel noise, muscle activity, and heart) were discarded *via* visual inspection with the help of plugin extensions in EEGLAB (i.e., ICLabel, DIPIT, and ADJUST). There were no significant differences in the number of bad channels or artifactual components between stroke patients and healthy controls. See Supplementary Table 2 for more details. EEG data acquisition and preprocessing of the LEMON dataset were extensively described in the previous studies (Babayan et al., 2019; Férat et al., 2020; Zanesco et al., 2020).

### Microstate-Based Analysis of Brain Dynamics

**FIGURE 1:**
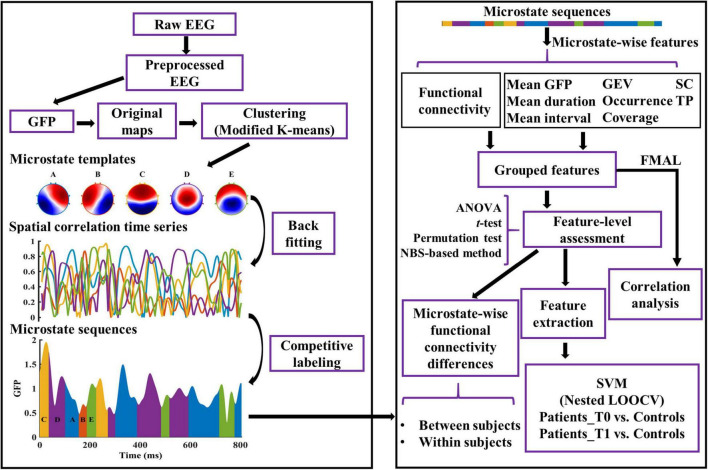
Graphical representation of the analysis method in the study. The microstate templates used in this study are common templates of patients and healthy controls. See “Materials and Methods” section for details. GFP, Global Field Power; GEV, Global Explained Variance; SC, Spatial Correlation metric; TP, Transition Probability; FMAL, Lower-extremity part of Fugl-Meyer Assessment; SVM, Support Vector Machine; LOOCV, Leave-One-Out Cross-Validation.

Microstate analyses were performed in MATLAB. Some steps were based on the Microstate toolbox (MST, version 1.0) (Poulsen et al., 2018) and Microstate^1^ (version 1.2). The complete analysis pipeline used in this study is shown in Figure 1. EEG data were first lowpass filtered (order: 208, transition width: 4.0 Hz, −6 dB cutoff frequency: 22 Hz) and then re-referenced to the common average before microstate analysis. Local maximal values (peaks) of the global field power (GFP) were extracted from each EEG recording. GFP was calculated as the standard deviation of the amplitude across all channels at each time point. EEG maps at GFP peaks are reliable representations of the topographic maps because of their high signal-to-noise ratio (Koenig et al., 2002). Considering the high uncertainty in the assignment of states for maps with low GFP, we discarded the bottom 15% of GFP peaks (Mishra et al., 2020). GFP peaks that were greater than three times the standard deviation were also excluded. EEG maps at the remaining GFP peaks (also called original maps) were used for further analysis.

#### Identifying Recording-Specific Microstate Templates

For each recording, a modified k-means clustering algorithm (ignoring the polarity) was used to find 2–10 (*k* = [2:10]) templates using the original maps (Murray et al., 2008; Poulsen et al., 2018). For each *k*, the clustering procedure was repeated 100 times and returned the templates of the run with the largest global explained variance (GEV). Meanwhile, the GEV and the value of the Krzanowski–Lai (KL) criterion (Krzanowski and Lai, 1988; Murray et al., 2008) were determined for each *k*.

#### Identifying Group-Specific Microstate Templates

To have equal contributions of microstates per participant in the group, each participant provided the same number of templates to the second modified k-means clustering (van de Ville et al., 2010; Nishida et al., 2013). For each group, the optimal number of templates at the group level (k*) was determined by the number of clusters corresponding to the maximum value of the mean of the normalized KL across all recordings in the group. The k* templates from each participant of the group were concatenated, and then k* clusters were determined by the modified k-means algorithm. The centroids of the k* clusters were the group-specific microstate templates.

#### Identifying Common Microstate Templates

In this study, the optimal number was five in both the stroke patients and healthy controls. To avoid systematic variances derived from the differences of group-specific microstate templates between patients and healthy controls, the common microstate templates were used. To get the common templates, the modified K-means method was utilized for the group-specific microstate templates of patients and controls. The common templates were labeled A–E according to their similarities to the templates in the previous research (Férat et al., 2020; Shi et al., 2020; Zanesco et al., 2020).

#### Back-Fitting of the Microstate Templates

The common templates were fitted back to all the EEG maps (not just the maps at the GFP peaks) of each preprocessed EEG recording. EEG map at each time point was assigned a label according to the map with which the template demonstrated the highest absolute spatial correlation. Thereafter, EEG maps were converted into microstate sequences. Temporal smoothing was then employed in the microstate sequence by changing the labels of small segments (<30 ms) to the next most likely microstate class until no microstate segment was smaller than 30 ms (Poulsen et al., 2018; Liu et al., 2020). Note that the first and last microstates in each EEG segment (caused by the removal of artifacts) were potentially truncated. Thus, these microstates were not taken into consideration when calculating the parameters of each microstate class.

#### Microstate-Wise Functional Connectivity

In this study, the functional connectivity of each microstate class was computed by a phase-based method (i.e., PLV) across all channel pairs for the alpha band (8–12 Hz). EEG data were first applied with surface Laplacian transform using the CSD toolbox (Kayser and Tenke, 2006) to overcome the volume conduction problem. The phase signal was then extracted by applying the Hilbert transform to the band-filtered EEG data, and 10% phase angles of each side were discarded due to the edge effects. The segments of the phase angles belonging to a particular microstate class were picked up and concatenated together. Thereafter, the microstate-wise functional connectivity over time across all channel pairs was calculated. For instance, PLV of microstate class *m* between two channels *i* and *j* is calculated by the formula:


$$P\,L\,V_{i,j}^{m}=\left|\frac{1}{N}\,\sum_{n=1}^{N}e^{-\text{i}\,\left(\varphi_{j\,n}-\varphi_{i\,n}\right)}\right|,$$

where *N* is the number of time points belonging to microstate *m*, and φ_*i*_, φ_*j*_ are phase angles from channels *i* and *j*.

#### Microstate Parameters

For each EEG recording, the microstate sequence and microstate spatial correlation time series (dimension: *#* microstate classes × *#* time points) were computed (see Figure 1). Subsequently, dynamic parameters per microstate class were estimated. *GEV*, which measures the percentage of variance explained for the EEG maps across all time points by each specific microstate template. *Mean duration*, defined as the average of the continuous length of time during which the EEG time series is determined to be a certain microstate class. *Occurrence*, the average number of occurrences per second of each microstate class. *Coverage*, defined as the percentage of total analysis time occupied by each microstate class. *Mean interval*, the average across all the lengths of time from the end of a particular microstate class to the start of the next same microstate class. *Mean GFP*, the average amplitude of GFP during each microstate class dominance. *Spatial correlation metric* (SC), the mean absolute correlation values of each microstate template with maps of a given microstate class. For example, SC_*AB*_ is the average absolute correlation coefficients between the template of microstate A and all the maps belonging to microstate B. *Transition probability* (TP), defined as the probability (observed transition probability minus expected transition probability) from each microstate class to another (Nishida et al., 2013). See Murray et al. (2008), Nishida et al. (2013), Khanna et al. (2015), and Michel and Koenig (2018) for the details of the interpretation of microstate parameters.

### Statistical Analysis

In the study, the significance level is 0.05. All statistical tests are two-sided tests. All the reported *p*-values were based on the non-parametric permutation method (Knijnenburg et al., 2009; Zalesky et al., 2010). For multiple comparisons, we reported the *p*-values corrected by the Bonferroni-Holm method, unless specified otherwise.

#### For Microstate-Wise Functional Connectivity

A method based on the network-based statistic (NBS) was constructed to control the family-wise error rate as massive univariate tests were performed in connection comparisons (Zalesky et al., 2010). Briefly, the NBS-based algorithm utilized in this study comprises the following steps. (1) Compute both *p*-value and test-statistic (e.g., *t*-value or correlation coefficient) maps resulting from the statistical test of all connections. (2) A threshold (*p*_*thr*_) was applied to the *p*-value map. Elements below the threshold were set to 1, and the others were set to 0. The resulting mask was applied to the test-statistic map. The sparse test-statistic map was divided into two parts according to the signs of the elements. MATLAB function *conncomp* was used to determine the connection components for each part. The connected components and the size (e.g., the sum of *t*-values or correlation coefficient) of each component were determined for each part. (3) Perform permutation tests (2,000 permutations). For each iteration, the labels were randomly shuffled. More specifically, for between-subjects designs, condition labels were randomly shuffled; for within-subjects designs, the signs of the differences of a feature for each subject between two conditions were randomly assigned; for Pearson correlations, the orders of the FMAL scores were randomly shuffled. Repeat steps (1) and (2). Then, calculate the sizes of the largest positive and negative components and store them in a matrix, respectively. Note that the observed connected components and their sizes were computed and stored before the permutation procedure. (4) After all iterations, two null distributions were obtained, namely the sizes of the largest positive and negative components. For each observed connected component, the *p*-value was computed as the following formula:


$$p=2\times \frac{sum\left(abs\left({\text{S}}_{\mathrm{null}}\right)>abs\left(s_{o\,b\,s}\right)\right)+1}{N+1},$$

where *N* is the number of permutations, *s*_*obs*_ is the size of the observed connected component, and ***S***_***null***_ is one null distribution chosen based on the sign of the observed connected component’s size. For difference or correlation analysis, computing the mean of the functional connectivity strength within the connected component (mean FCSCC) is one way to reduce the feature dimension.

#### For Microstate Parameters

For patients at T0/T1 vs. healthy controls, for each microstate parameter, we conducted a two-way mixed ANOVA, with Group, Microstate Class/Spatial correlation Pair/Transition Pair as factors. Accordingly, for patients at T0 vs. patients at T1, for each microstate parameter, we conducted a two-way repeated ANOVA. If the interaction effect was significant, *post hoc* permutation tests were used for pairwise group comparisons for each level of the Microstate Class/Spatial correlation Pair/Transition Pair factor.

For ANOVAs above, the sphericity, normality, and homogeneity of variance were assessed by the Mauchly’s test, Kolmogorov-Smirnov test, and Levene’s test, respectively. If the assumption of sphericity was violated, the degrees of freedom were corrected using Greenhouse-Geisser correction if ε < 0.75, otherwise, using Huynh-Feldt correction. If the assumption of normality or homogeneity of variance was violated, pairwise group comparisons were directly performed using permutation tests. Permutation tests for two samples and paired samples were computed with 100,000 replications using the *t*-value as a measure of group difference. We also performed correlation analysis between microstate parameters and FMAL scores. *p*-values were calculated by permutation test with 100,000 replications using Pearson correlation coefficient as the test statistic. The 95% confidence interval (CI) was generated by the bootstrap method (10,000 bootstrap data samples; CI = bias corrected and accelerated).

### Classification

We employed SVM models to assess whether microstate-wise features could provide reliable signatures of stroke. Due to the massive number of features, the microstate parameters, including mean FCSCC, which were significantly different between stroke and health at both T0 and T1 were retained for further analysis. SVM models were conducted with the Python package *scikit-learn*. We constructed the SVM classifiers using linear and radial basis function (RBF) kernels, respectively. To avoid an optimistically biased evaluation of the model performance, the nested leave-one-out cross-validation (nested LOOCV) was utilized to evaluate the tuned SVM models. Moreover, the *scikit-learn pipeline* of the preprocessing steps was employed to prevent data leakage in the cross-validation and hyper-parameter tuning procedures. For each iteration of the outer LOOCV loop, one sample was split as the test dataset and the rest as the training dataset. We first scaled the features to have mean 0 and variance 1 and then used principal component analysis (PCA) for feature reduction (retaining 95% variance) before training each model. Then, in the inner LOOCV loop, select the best hyperparameters based on grid search hyperparameter optimization and refit a model with the entire training dataset with the parameters. This model then was used to predict the test dataset of the outer LOOCV loop. After all iterations of the outer loop, the prediction results of all samples were collected, and then the model performance was evaluated by the receiver operating characteristic (ROC) curve and the area under the curve (AUC) of the ROC.

### Statistical and Visualization Tools

All ANOVAs were carried out in IBM SPSS Statistics 26 (SPSS IBM, Armonk, New York, United States). The other statistical analysis was conducted with custom MATLAB scripts. Schemaball plots were made with some modifications to the schemaball project.^2^ Networks with EEG electrodes as nodes (Koessler et al., 2009) were created by BrainNet Viewer software (Xia et al., 2013). Violin plots were generated by OriginPro 2022 Beta2 (OriginLab Corporation, Northampton, MA, United States). Other figures were created using custom MATLAB scripts.

## Results

### Patients Findings

The sample demographics and clinical information are summarized in Table 1. After 10 sessions of training, patients (*N* = 31) showed significantly increased FMAL scores (*p* = 1.000e-5) compared to the baseline (Supplementary Figure 1B). Moreover, patients with right-sided stroke (*N* = 12) presented higher FMAL scores than those with left-sided stroke, but the differences were not significant at both T0 (*p* = 0.131) and T1 (*p* = 0.161). On average, the time poststroke at T0 was 65.9 days (*SD* = 39.7 days) and patients with left-sided stroke showed a slightly shorter time than patients with right-sided stroke (*p* = 0.419). In this study, only six patients had a hemorrhagic stroke and seven patients were female. Considering the reliability of the results, no further statistical analysis was conducted to compare the differences between stroke-type subgroups or between gender subgroups.

### Microstate Templates

**FIGURE 2:**
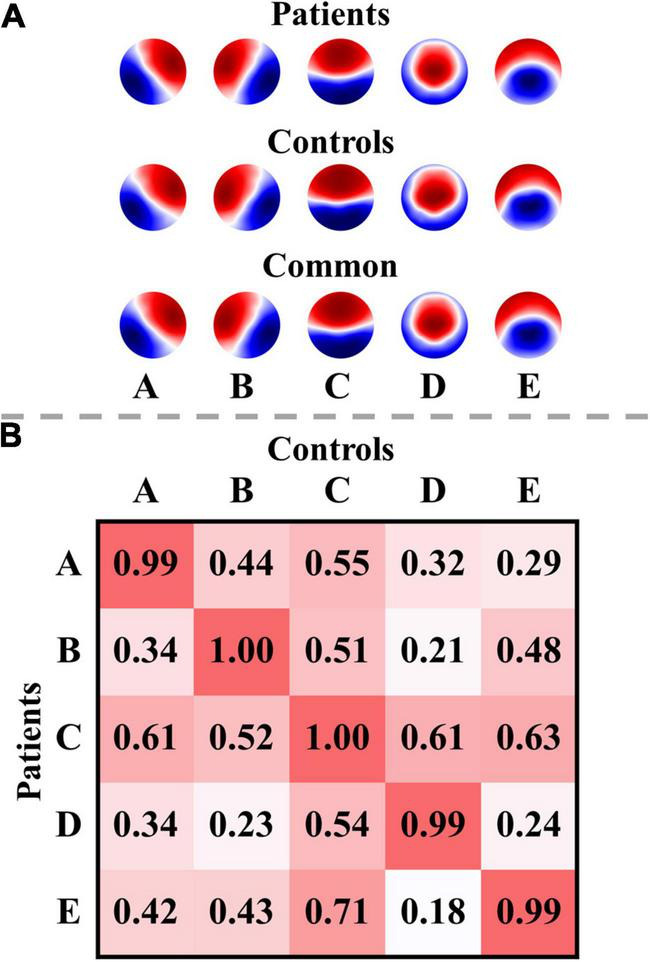
Microstate templates and spatial correlations. **(A)** Microstate templates. **(B)** Spatial correlations between the microstate templates of patients and controls. Polarity was ignored when computing the correlations. The templates of the patients were calculated from all their EEG data at T0 and T1.

The optimal number of microstate classes was five for both the patients and controls. The five microstate templates of the patients and controls were provided in Figure 2A. Interestingly, the microstate templates of patients and controls were highly similar (all *rs* > 0.99), as shown in Figure 2B. Nevertheless, to reduce the potential systematic variances caused by the template differences between patients and controls, the common microstate templates were utilized in further analysis. The five common microstate templates were labeled A–E according to their similarities to the templates reported in the previous large sample size study (Zanesco et al., 2020). On average, the templates explained 80.52, 80.82, and 79.86% of the GEV (fitting to maps at GFP local maxima) for patients at T0, patients at T1, and healthy controls, respectively.

### Microstate-Wise Functional Connectivity

**FIGURE 3:**
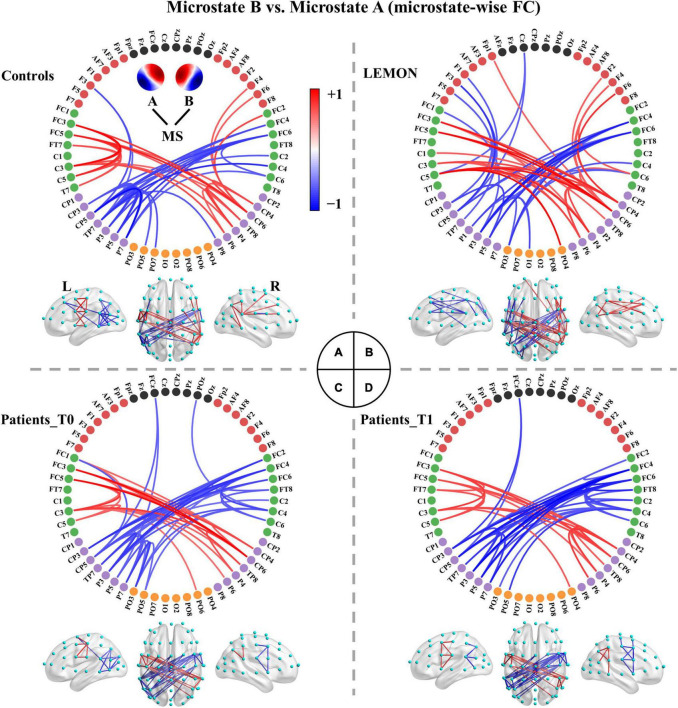
Comparisons between functional connectivity of microstates A and B for the four groups. **(A)** Healthy controls. **(B)** LEMON. **(C)** Patients at T0. **(D)** Patients at T1. For visual clarity, only the top 50 connections (according to absolute *t*-values) in the connected components with significant differences (*p* < 0.05, NBS-based method corrected) are displayed in the figure. The red color indicates greater connectivity for microstate B vs. microstate A, while the blue color indicates greater connectivity for microstate A vs. microstate B. Color depth represents the size of the connection difference (*t*-value). The complete connected components are provided in the first column of Supplementary Figure 3.

The functional connectivity associated with each microstate class is displayed in Supplementary Figure 2. Intuitively, the functional connectivity of microstate C appeared to be stronger compared to the other microstate classes. The microstate-wise connectivity seemed similar to each other due to the masking of strong short-range connections. However, the comparison results (*p*_*thr*_ = 0.001) demonstrated significant differences between functional connectivity of microstate classes (Supplementary Table 3). For convenience, FC_*X*_ refers to the functional connectivity of microstate X. To further evaluate the stability and robustness of the results, an independent EEG dataset of healthy subjects, LEMON, was introduced in the present study. Briefly, after NBS-based correction, all but two of the comparison pairs of microstate-wise functional connectivity were significant (*ps* < 0.05) for all four groups (the smallest *p*-value of FC_*C*_ < FC_*A*_ of controls *p* = 0.096 and FC_*C*_ < FC_*B*_ of patients at T1 *p* = 0.099). Strikingly, the patterns of differences between microstate-wise functional connectivity were highly consistent and stable for all groups, as provided in Figure 3 and Supplementary Figures 3–12. For instance, as illustrated in Figure 3 and Supplementary Figure 3, for the comparison between FC_*A*_ and FC_*B*_ of each group, there was a significant connected component for FC_*A*_ > FC_*B*_ (*p* = 0.001) and FC_*A*_ < FC_*B*_ (*p* = 0.001) respectively. FC_*A*_ showed a stronger strength of PLV mainly involving interhemispheric connections between the left parietal and right frontocentral-central areas, and intrahemispheric connections between the left parietal and left frontal areas and connections within the left parietal/right frontocentral areas compared to FC_*B*_. However, for FC_*A*_ < FC_*B*_, there was a symmetrical pattern about the anterior-posterior axis relative to FC_*A*_ > FC_*B*_. Microstate templates A and B exhibited a left-posterior right-frontal orientation and right-posterior left-frontal orientation, respectively (see Figure 3A). Interestingly, the patterns of the differences between FC_*A*_ and FC_*B*_ also revealed the corresponding symmetry and harmony.

**FIGURE 4:**
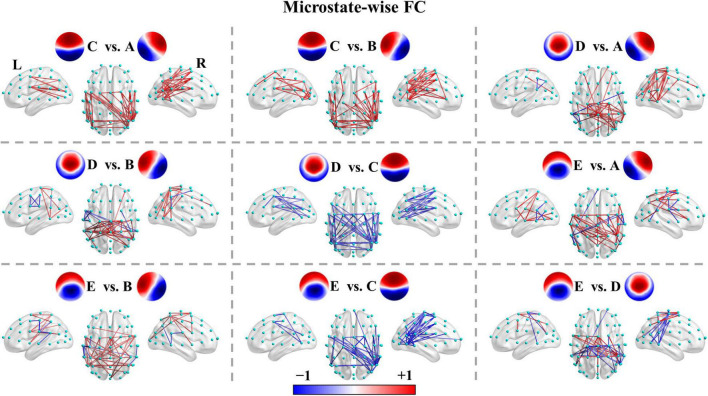
Microstate-wise functional connectivity comparisons in healthy controls. For X vs. Y, the red color indicates greater connectivity for microstate X vs. microstate Y, while the blue color indicates greater connectivity for microstate Y vs. microstate X. Color depth represents the size of the connection difference (*t*-value). For visual clarity, only the top 50 connections (according to absolute *t*-values) of the connected components with significant differences (*p* < 0.05, NBS-based method corrected) are displayed in the figure. The complete significant connected components of the four groups are shown in the first column of Supplementary Figures 4–12.

We recapitulated the unique connectivity pattern of each microstate class based on the patterns of differences between the functional connectivity of one microstate class and the other microstate classes (Figure 4). See Supplementary Figures 3–12 for more details. FC_*A*_ showed stronger connections mainly between the left parietal (e.g., CP3, CP5, P1, P3, P5, P7) and right frontocentral-central (e.g., FC2, FC4, FC6, FT8, C4, C6) areas and within the left parietal/right frontocentral areas. Accordingly, FC_*B*_ displayed greater connections mainly between the right parietal (CP4, CP6, P2, P4, P6, P8) and left frontocentral-central (e.g., FC1, FC3, FC5, FT7, C3, C5) areas and within the right parietal/left frontocentral areas. FC_*C*_ exhibited a stronger strength of PLV mainly involving bilateral frontocentral-parietal (e.g., FC1, FC3, FC5, P3, P5, P7; FC2, FC4, FC6, P4, P6, P8) connections and interhemispheric parietal (e.g., P5, P6, P7, P8) connections. The pattern of FC_*C*_ displayed a U shape and was symmetrical about the anterior-posterior axis like template C. Moreover, FC_*D*_ presented greater connections mainly within the central areas (e.g., Cz, C1, C2, CPz, CP1, CP2). Furthermore, FC_*E*_ showed stronger connections mainly between the left frontocentral (e.g., FCz, FC5, FT7)/left parietal (e.g., CPz, CP1, P3) and right frontocentral (e.g., FC6, FT8) areas/right parietal areas (e.g., CP2, P4) and between middle line channels (e.g., FCz, Cz, CPz, Pz). In sum, these results suggest each microstate class has a distinct connectivity preference and corresponds to a unique functional connectivity pattern. Intriguingly, the connectivity pattern of a microstate class appeared to reflect its template.

For patients with left- or right-sided stroke, the connectivity patterns were weakened to varying degrees, but the main skeleton of the patterns seemed to be preserved (see Supplementary Figures 13–16). Next, we examined the difference in microstate-specific connectivity between the patients at T0 and patients at T1 (*p*_*thr*_ = 0.01). Patients at T1 showed weaker connections mainly in the anterior regions in FC_*A*_ (*p* = 0.176) and stronger long-range connections (e.g., frontal-parietal connections) in FC_*D*_ (*p* = 0.075), but none connected components survived the NBS-based correction (see Supplementary Figure 17). We then examined the differences between patients at T0/T1 and controls (*p*_*thr*_ = 0.01). Once again, none of the connected components survived the correction.

### Microstate Parameters

#### Two-Way ANOVAs

For patients at T0 vs. patients at T0, two-way repeated-measures ANOVAs showed non-significant Time × Microstate Class interaction effects for all microstate features. The main effects of Time were also non-significant. Therefore, *post hoc* comparisons were not further performed.

For patients at T0 vs. healthy controls, two-way mixed ANOVAs showed significant Microstate Class × Group interaction effects for occurrence [*F*(3.721, 193.488) = 5.728, *p* = 3.268e-4, $\eta_{p}^{2}$ = 0.099], coverage [*F*(2.370, 123.251) = 9.228, *p* = 7.073e-5, $\eta_{p}^{2}$ = 0.151], and mean GFP [*F*(2.965, 154.176) = 7.741, *p* = 8.092e-5, $\eta_{p}^{2}$ = 0.130]. The analysis also revealed significant Spatial Correlation pairs × Group interaction for SC [*F*(4.515, 234.781) = 6.090, *p* = 5.149e-5, $\eta_{p}^{2}$ = 0.105], and Transition pairs × Group interaction for TP [*F*(6.715, 241.758) = 3.591, *p* = 0.001, $\eta_{p}^{2}$ = 0.091]. For patients at T1 vs. healthy controls, two-way mixed ANOVAs demonstrated significant Microstate Class × Group interaction effects for occurrence [*F*(3.533, 183.693) = 3.042, *p* = 0.025, $\eta_{p}^{2}$ = 0.055], coverage [*F*(2.148, 111.693) = 5.446, *p* = 0.004, $\eta_{p}^{2}$ = 0.095], and mean GFP [*F*(2.712, 141.039) = 5.059, *p* = 0.003, $\eta_{p}^{2}$ = 0.089]. There was also significant Spatial Correlation Items × Group interaction for SC [*F*(4.418, 229.711) = 4.957, *p* = 4.710e-4, $\eta_{p}^{2}$ = 0.087], and Transition pairs × Group interaction for TP [*F*(5.834, 204.174) = 2.795, *p* = 0.013, $\eta_{p}^{2}$ = 0.074]. The two-way mixed ANOVAs for GEV, duration and interval at both T0 and T1 had one combination of two factors severely violating the homogeneity of variances hypothesis. For these three microstate parameters, pairwise comparisons for each microstate class between patients and controls were performed by permutation test directly.

#### *Post hoc* Comparisons

##### Global Explained Variance (GEV)

**FIGURE 5:**
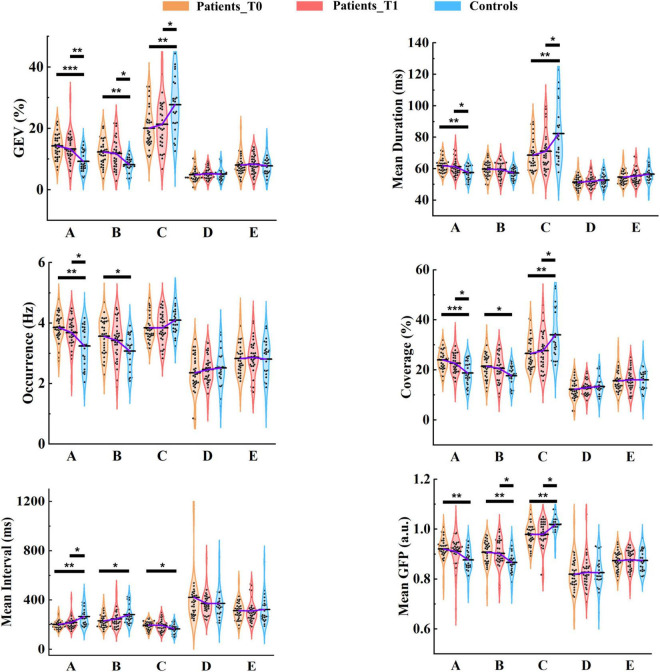
Results of microstate parameter analysis for patients and controls. *Post hoc* pairwise comparisons (patients at T0 vs. controls; patients at T1 vs. controls) were performed for GEV, mean duration, occurrence, coverage, mean interval, and mean GFP. The means of the three groups for each microstate class are linked by the solid purple line. *p < 0.05; **p < 0.01; ***p < 0.001.

On average, the GEV for each microstate class ranged from 4.84 to 20.05% in patients at T0, from 5.22 to 21.31% in patients at T1, and from 5.06 to 27.74% in healthy controls. Group average statistics (± *SD*) for microstate parameters are provided in Supplementary Table 4. It is worth noting that microstate template C explained more variance relative to other templates (all *ps* < 0.05), and was the only template that explained more than 15% variance for each group (Figure 5). Furthermore, microstate C showed a significantly higher GEV in healthy controls compared to patients at T0 (*p* = 0.002) and T1 (*p* = 0.027), whereas microstates A and B showed significantly lower GEV in healthy controls compared to patients at T0 (*p* = 1.000e-4, *p* = 0.001) and T1 (*p* = 0.006, *p* = 0.010). For microstates D and E, there was no significant difference between the patients and controls. The detailed statistical results are summarized in Supplementary Tables 5, 6.

##### Mean Duration

On average, the mean duration for each microstate class lasted between 51.42 and 68.63 ms in patients at T0, between 52.04 and 71.04 ms in patients at T1, and between 52.81 and 82.24 ms in healthy controls. The mean duration of microstate C was predominant and significantly longer than that of the other microstate classes (all *ps* < 0.001) in healthy controls. However, the predominant pattern of microstate C was considerably weakened in patients. No significant differences between microstates A and C were observed in patients at either T0 (*p* = 0.898) and T1 (*p* = 0.300). Microstate C showed a significantly longer duration in healthy controls compared to patients at T0 (*p* = 0.003) and T1 (*p* = 0.040). In contrast, The mean duration of microstate A was significantly longer in patients at T0 (*p* = 0.003) and T1 (*p* = 0.045) relative to healthy controls.

##### Occurrence

In healthy controls, on average, microstate C occurred 4.09 times (*SD* = 0.46) per second, and microstates A, B, D, and E occurred 3.24 (*SD* = 0.65), 3.07 (*SD* = 0.56), 2.51 (*SD* = 0.61), and 2.80 (*SD* = 0.66) times per second, respectively. As shown in Figure 5, in patients at T0 and T1, the occurrences of microstates A, B, and C were all about 4 times per second on average. More specifically, microstates A and B occurred more frequently in patients at T0 (*p* = 0.001, *p* = 0.015) and T1 (*p* = 0.037, *p* = 0.191) compared to healthy controls, and the predominant pattern of microstate C in controls disappeared in patients.

##### Coverage

There were significant differences between patients at T0 and controls in microstates A (*p* = 1.800e-4), B (*p* = 0.012), and C (*p* = 0.005), and between patients at T1 and controls in microstates A (*p* = 0.023) and C (*p* = 0.043). The dominant pattern of microstate C also existed in the coverage, especially in healthy controls.

##### Mean Interval

For each group, the mean interval of microstate C was the shortest among all microstate classes (all *ps* < 0.001). For patients at T0 vs. healthy controls, the mean interval was significantly shorter in microstates A (*p* = 0.001) and B (*p* = 0.018). In contrast, the opposite pattern was observed in microstate C (*p* = 0.021). At T1, only the mean interval of microstate A (*p* = 0.031) was still significant between patients and controls.

##### Mean Global Field Power (GFP)

Microstate C exhibited a significantly stronger GFP compared to the other microstate classes (all *ps* < 0.001) in healthy controls, but for patients, this pattern was weakened. Microstate C presented significantly stronger GFP in healthy controls relative to patients at both T0 (*p* = 0.001) and T1 (*p* = 0.015), but microstates A (*p* = 0.001, *p* = 0.116) and B (*p* = 0.003, *p* = 0.049) showed greater GFP in patients at T0 and T1 compared to healthy controls.

Of Note, there were no differences in the summed/average microstate parameters across the five microstate classes between patients at T0/T1 and healthy controls. Moreover, although the differences between the patients at T0 and T1 were not significant, we observed that microstate parameters at T1 of patients were closer to those of healthy controls compared to T0 at the group level.

##### Spatial Correlation Metric (SC)

As demonstrated in Supplementary Figure 18, intuitively, all three groups followed a similar pattern in SC. However, patients presented higher spatial correlations in SC_*AA*_, SC_*AB*_, SC_*BA*_, SC_*BB*_, SC_*CC*_, SC_*DD*_, and SC_*EE*_ at both T0 and T1 compared to healthy controls (see also Supplementary Table 7). SC_*AB*_, SC_*BA*_, and SC_*BB*_ were still signifificantly higher for patients at both T0 and T1 relative to healthy controls after correction for 25 times comparisons.

##### Transition Probability (TP)

We observed that TP_*BC*_, TP_*DC*_, and TP_*EC*_ significantly decreased, and TP_*BA*_ and TP_*EA*_ significantly increased in patients at T0 compared to controls, but none survived Bonferroni-Holm correction for 20 times comparisons. At T1, similar patterns were also observed, but to a lesser degree (Supplementary Figure 19).

We conducted further analysis to examine whether the microstate parameters differed between patients with left- and right-sided stroke. No significant differences were observed in any of the microstate parameters at either T0 or T1. The results of mean duration and occurrence are presented in Supplementary Figure 20.

### Classification

**FIGURE 6:**
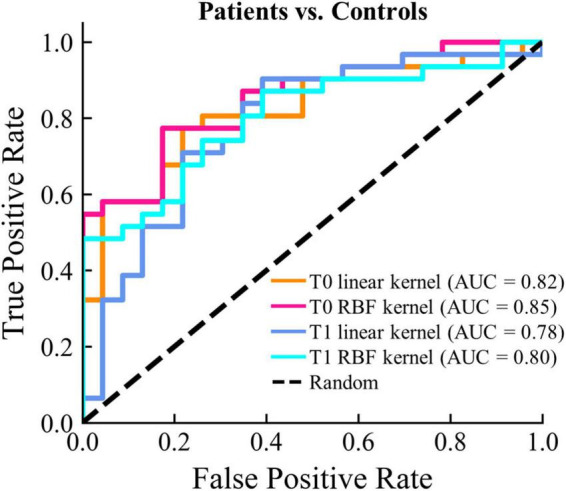
Classifications results between patients and controls. The ROC curve and the corresponding AUC for each classification model are displayed.

To evaluate the ability to discriminate between patients and healthy controls using only the microstate features, we constructed SVM models with different kernel types. It is worth noting that we only sought to assess whether there was a basic discriminating ability of the microstate parameters instead to find the best-performing model through complex feature selection and parameter tuning procedures. For feature selection, we first selected microstate features that were both significantly different between patients and controls at T0 and T1. PCA was utilized to further reduce the dimensions of the features and retained only the 95% variance. To avoid the optimistically biased evaluation of the model performance, the nested LOOCV was used, and the *scikit-learn pipeline* was adopted to prevent data leakage in the cross-validation and hyper-parameter tuning procedures. As shown in Figure 6, AUCs were larger than 0.80 for patients at T0 vs. controls with both the linear and RBF kernels. AUCs were slightly lower (linear: 0.78; RBF: 0.80) for patients at T1 vs. controls. In addition, the AUC of the SVM model with RBF kernel was slightly higher at both T0 and T1. These results demonstrate that moderate discrimination between patients and healthy controls can be achieved using only the microstate features.

### Correlation Analysis

**FIGURE 7:**
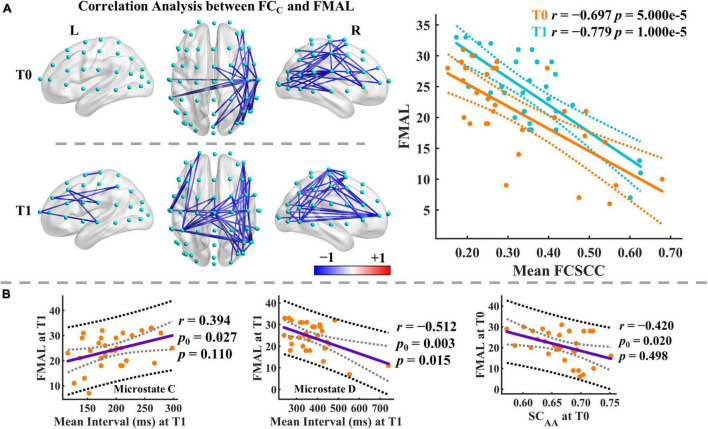
Results of correlation analysis. **(A)** Correlation analysis between the functional connectivity of microstate C and FMAL score. Only the significant connected components are displayed. Mean FCSCC refers to the mean of the functional connectivity strength within the significant connected component. The blue color indicates negative correlations between the connectivity of microstate C and the FMAL score. Color depth represents the size of the correlation coefficient. Dashed lines imply the 95% confidence intervals for the regression estimates. Pearson correlation coefficient (*r*) and the corresponding permutation-based *p*-value are given. **(B)** Correlation analysis between microstate parameters and FMAL score. The black and gray dashed lines indicate the 95% prediction and 95% confidence intervals, respectively. Pearson correlation coefficient (*r*) and the corresponding permutation-based *p*-value are given. *p*_0_ is the uncorrected *p*-value.

We finally examined the correlations between the microstate features and the FMAL scores. We also used the NBS-based method for multiple comparison correction for correlations between microstate-specific connectivity and FMAL score (*p*_*thr*_ = 0.005). Broadly speaking, there was a significant connected component with negative correlations at T0 and T1 for most microstate classes (see Supplementary Figure 21). At T0, the significant connected components mainly involved the right hemisphere. More connections involving the left hemisphere and middle line channels were included in the significant connected components at T1. For instance, the distribution of the connected component of microstate C mainly affected the connections between the right central (e.g., C2, C4, C6, T8) and right posterior/frontal areas at T0 and between the left central (C1, C3, C5, CP3)/right frontal-central (AF8, FC2, FC4, C2, C4) and middle line (FCz, Cz)/right posterior areas at T1 (Figure 7A).

Moreover, we identified significant correlations between the mean FCSCC of microstate C and FMAL score at both T0 (*r* = −0.697, *p* = 5.000e-5, 95% CI [−0.830, −0.452]) and T1 (*r* = −0.779, *p* = 1.000e-5, 95% CI [−0.895, −0.546]). FMAL score was moderately correlated with microstate parameters (e.g., occurrence, coverage) mainly in microstates C and D at T1 and similar relationships were observed at T0, but to a lesser degree (see Supplementary Table 8). Intriguingly, the parameters in microstates C and D showed opposite correlation patterns with the FMAL score. For instance, at T1, the mean interval was positively correlated with the FMAL score in microstate C (*r* = 0.394, *p*_0_ = 0.027, *p* = 0.110, 95% CI [0.046, 0.645]) but negatively correlated with the FMAL score in microstate D (*r* = −0.512, *p*_0_ = 0.003, *p* = 0.015, 95% CI [−0.755, −0.045]) (see Figure 7B). *p*_0_ is the uncorrected *p*-value. On the other hand, there were moderate correlations between the FMAL score and SC at T0 (e.g., SC_*AA*_, SC_*AB*_, SC_*AD*_, SC_*AD*_, SC_*AE*_). For example, SC_*AA*_ at T0 was negatively correlated with the FMAL score (*r* = −0.420, *p*_0_ = 0.020, *p* = 0.498, 95% CI [−0.650, −0.087]), but none of them was significant after the Bonferroni-Holm correction for multiple comparisons (see Supplementary Table 9).

## Discussion

The brain is a complex dynamically distributed information processing system with stable structural connections that support time-varying information transfer between brain regions (Park et al., 2021). Yet, after the stroke, it remains unclear how the dynamic neural signals of the brain are altered due to damage to the neural tissue of the brain, together with changes in receptor distribution. Understanding the mechanisms underlying brain dynamics and the alterations in brain dynamics after brain injury is a cutting-edge question in neuroscience and neurological rehabilitation (Comsa et al., 2019; Bonkhoff et al., 2020; Zanesco et al., 2020; Bai et al., 2021a; Ploner and Tiemann, 2021). We explored the phase-coupling pattern of each microstate class and the spatiotemporal dynamics of the brain in stroke patients and healthy controls using microstate-based analysis. Our results demonstrate that each microstate class corresponds to a distinct pattern of functional connectivity, and the results of different datasets are highly similar from a macroscopic point of view. On the other hand, stroke patients showed significant changes in almost all types of microstate parameters compared to the healthy population. In addition, some microstate-related features were significantly associated with lower limb motor function. Together, our work demonstrates the close association between microstates and functional connectivity, and the importance of studying altered neural features of the brain after stroke from a dynamic perspective.

We found that the optimal number of microstate classes was five in both healthy controls and stroke patients, and the microstate topographies were highly similar between the groups. There is no consensus on how to determine the optimal number of microstate classes (Custo et al., 2017; Michel and Koenig, 2018; Gui et al., 2020). The number of microstate classes was determined or set to four in many previous experimental and clinical studies (Michel and Koenig, 2018). Although the optimal number is associated with the dataset used and the selection of the determination criterion, a low number of microstate classes may leave a large amount of data variance unexplained (for all maps, not only those at GFP peaks), as well as the risk of merging different classes (Custo et al., 2017). Here, the microstate templates were highly similar to those reported in previous studies (Shi et al., 2020; Zanesco et al., 2020, 2021). Yet, only one previous study, to our knowledge, used the complete microstate analysis in stroke patients (Zappasodi et al., 2017). In the study by Zappasodi and colleagues, the optimal number determined using the KL criteria and cross-validation (CV) criteria was four, and the microstate templates of healthy controls differed from those of stroke patients (patients with the lesion in the left/right hemisphere). Compared to our results, one possible reason for the discrepancy is that they used a different method for determining the optimal number of microstate classes and utilized 19-channel EEG data from acute stroke patients, whereas our study used 60-channel EEG data from subacute stroke patients.

Having computed the common microstate templates, we investigated the microstate-specific functional connectivity in the sensor space. Many studies have explored the source localization of microstates, and the association between the microstates and RSNs (Britz et al., 2010; Yuan et al., 2012; Custo et al., 2017; Milz et al., 2017; Michel and Koenig, 2018). Currently, only limited studies have sought to uncover the relationship between resting EEG microstates and their corresponding functional connectivity (Comsa et al., 2019). The functional connectivity patterns underlying each microstate class remain unclear. Moreover, a popular approach for studying brain dynamics in EEG and fMRI is dynamic functional connectivity (Hutchison et al., 2013; Allen et al., 2014; Hansen et al., 2015; Kabbara et al., 2017; O’Neill et al., 2018; Ploner and Tiemann, 2021). The major limitation of the dynamic functional connectivity analysis based on the sliding window approach is the need to manually select a fixed window length. This means that the signals under this window length may not belong to the same brain state. Microstate-specific functional connectivity analysis is similar in nature to dynamic functional connectivity, but in this study, the connectivity template for each microstate class is computed by the data belonging to this microstate class. More specifically, microstate analysis provides prior knowledge for computing the state-specific connectivity templates. Our analysis highlighted that each microstate class is associated with a unique phase synchronized pattern, and the functional connectivity pattern seems to reflect the character of the corresponding microstate template. These findings were highly consistent across patients and healthy controls groups and were replicated in an independent dataset. The connectivity patterns were weakened to varying degrees in patients with left- or right-sided stroke, but the main skeleton of the patterns seemed to be preserved. Moreover, there were no significant differences between patients and controls in microstate-wise functional connectivity. These results indicate that the similarity of connectivity patterns between groups may explain the similarity of the microstate templates. Note that the similarity of connectivity patterns was not a sufficient and necessary condition for no significant differences between healthy controls and patients. Due to the large heterogeneity of the patients, the effect size may be reduced at the channel-level connection. Importantly, these findings appear to prove that flipping the data in some studies to unify the lesions to one side may have an unpredictable effect on the results. For example, microstates A and B appear to be exchanged after the data are flipped. Our study extends and refines our understanding of microstates. Microstate analysis has the potential to study dynamic functional connectivity from a new perspective.

Due to the brain damage introduced by stroke, the cognition and behavior of the patients were impaired. We found significant changes in microstate parameters between patients and controls. The most striking results were in the parameters of microstates A, B, and C. In patients, microstate C explained less variance, was of lower mean GFP, had a shorter duration, longer mean interval, occupied less time and occurred less frequently compared to healthy controls. In contrast, microstates A and B exhibited the opposite pattern relative to microstate C. Importantly, no difference was observed in the summed parameters across five microstates between the patients and healthy controls. On the other hand, functional connectivity of microstate C showed stronger connectivity in a large connected component compared to other microstate classes. Partially in line with the findings of dynamic functional connectivity (Bonkhoff et al., 2020; Wang et al., 2020), stroke patients prefer to spend more time in states with low levels of connectivity. After brain injury, the alpha power is generally considerably decreased (Edlow et al., 2021). Herein stroke patients showed a decreased mean duration of microstate C. This may be partly explained by the positive association between the duration of microstate C and alpha power (Croce et al., 2020). Moreover, our study did not find significant differences between patients with left-sided and right-sided stroke in all microstate parameters, whereas the duration, occurrence, and coverage in microstates C and D significantly differed between patients with left-sided and right-sided stroke in a previous study (Zappasodi et al., 2017). A possible explanation is that the microstate templates between the two groups were different in the prior study and the poststroke time of the patients was different from that of our study. Further studies are needed to investigate this issue. In addition, stroke patients showed clearer patterns of changes in spatial correlation metrics relative to healthy controls. Briefly, the mean absolute correlation coefficients between the maps belonging to each microstate class and the corresponding template increased. The previous study has demonstrated that there were gradual map changes from one microstate to another (Mishra et al., 2020). These results suggest that after stroke the signal variability over time is diminished, and the microstate segregation seems to increase. One potential explanation is that the disruption of structural and functional connections after the stroke affects brain information integration and transmission, and limits the ability to dynamically configure the brain network. Moreover, the stroke-related alterations were also reflected in transition probability. TP_*BC*_, TP_*DC*_, and TP_*EC*_ occurred less frequently, while TP_*BA*_ and TP_*EA*_ were more likely to occur compared to controls. Overall, the results of this study suggest that the original dynamic balance between microstates is broken and seems to reflect the compensation and reconfiguration of network dynamics after stroke and that the reconfiguration of temporal dynamics is state-selective.

Previous studies have investigated the association between microstates and RSNs (Britz et al., 2010; Custo et al., 2017; Milz et al., 2017; Brechet et al., 2019; Zoubi et al., 2020). However, no complete consensus has yet been reached. Prior study (Britz et al., 2010) suggested that microstate classes and the corresponding most relevant RSNs were: the auditory network (microstate A), visual network (microstate B), saliency network (microstate C), and attention network (microstate D). However, other studies suggested that microstate C reflects a portion of the default mode network (Seitzman et al., 2017), and microstates A and B considerably overlap with the somatomotor network (Zoubi et al., 2020). An increase in the coverage and occurrence of microstate B was observed in certain cognitive tasks with direct visual input (Zappasodi et al., 2019), and an increased presence in microstate C and decreased presence in microstate D was observed in schizophrenia (da Cruz et al., 2020). Moreover, One microstate study demonstrated that spontaneous brain activity could encode detailed information about motor control (Pirondini et al., 2017). Given the complex relationships of microstate, cognition, and behavior, it is arbitrary to reduce microstates to specific functions with current knowledge.

Despite the high heterogeneity of stroke patients, we demonstrated the ability to achieve a moderate level of discrimination between stroke patients and healthy controls using only microstate features. Although stroke patients did not show differences in microstate parameters between T0 and T1, microstate parameters at the group level were closer to those of controls after the 2-week rehabilitation therapy. Finally, we evaluated the associations between microstate parameters and the clinical score of lower limb motor function. For each microstate class, the connectivity in a large connected component was negatively correlated with the FMAL score at both T0 and T1. These connected components mainly involved connections between sensorimotor areas and frontal/posterior regions and connections within sensorimotor areas. The negative correlation between static functional connectivity and FMAL was also observed in a recent study (Hoshino et al., 2021). In addition, the microstate dynamics may reflect the selective inhibition of specific intra-cortical regions (Milz et al., 2017). Patients with eyes closed were in an internally focused state, under the functional competition between different networks, weaker phase synchronization may reflect a greater potential for movement. Moreover, some spatial correlation metrics (e.g., SC_*AA*_) were negatively correlated with the FMAL score at T0. At T1, the correlations between the FMAL score and other parameters (e.g., occurrence, coverage, mean interval) of microstates C and D increased. Brain dynamics may be modulated by rehabilitation therapy, and resting-state brain dynamics can encode information reflecting motor ability. Furthermore, microstates C and D exhibited opposite patterns of association with the FMAL score in occurrence, coverage, mean interval, duration, etc. This may be due to the different functional connection patterns underlying microstates C and D. Together, these findings provide the first evidence that the microstate-related features are associated with the lower limb function status. Further studies are needed to validate these results and elucidate these correlations.

Several considerations should be taken into account. First, for patients in this study, we only focused on the stroke patients in the subacute phase. Given the sample size of the study, the analysis in more specific groups (e.g., grouping according to gender, stroke type, or age) was not performed. In addition, because of the large heterogeneity of stroke patients (e.g., age, lesion location, poststroke time, stroke type, and treatment), the results in this study are not sufficient to be applicable across the entire spectrum of stroke patients and need to be interpreted with caution. In addition, information on age- and gender-related changes of microstate dynamics is sparse, and there are huge discrepancies in the results of different studies, especially on gender (Tomescu et al., 2018; Zanesco et al., 2020). Second, although the differences in the microstate templates between patients and controls were small, we used the common templates to minimize the systematic errors. Due to the possible differences in the number of microstate classes or microstate templates, comparisons between studies need to be made with caution. Third, for microstate-wise functional connectivity, we only investigated the results in the alpha band. This is because the study of phase synchronization using the Hilbert transform approach requires the use of narrowband signals (Lachaux et al., 1999), and microstates are predominantly generated by intracortical sources in the alpha band (Milz et al., 2017). In addition, functional connectivity is based on the phase synchronization method, and phase- and amplitude-coupling patterns may reflect partly distinct neuronal mechanisms (Siems and Siegel, 2020). Fourth, a large number of dependent measures and multiple comparisons were conducted in this study. After strict *p*-value correction, there is a risk that the true effects will not be identified as statistically significant. The original *p*-values of the results are provided in Supplementary Material. To sum up, given the limitations of this study and the unsolved problems in this field, more meticulous and large sample size studies are needed in the future to validate and expand this research in the hope of gaining a deeper and more comprehensive understanding of brain dynamics.

## Conclusion

In a nutshell, our results provide a novel perspective on one of the major issues in neuroscience and neurological rehabilitation: the connectivity patterns underlying different functional states and how the neural dynamics are altered in brain injury patients (e.g., stroke) (O’Neill et al., 2018; Bai et al., 2021a; Bian et al., 2021). Centered on this issue, we proposed and implemented a microstate-based method to investigate the phase synchronization pattern underlying each microstate class and the alterations of microstate dynamics on the order of milliseconds in stroke patients. The results suggested that each microstate class was associated with a specific functional connectivity pattern, and the findings were highly consistent across different datasets. On the other hand, most microstate parameters (e.g., mean duration, occurrence, coverage, mean interval) in patients significantly differed from healthy controls in microstates A, B, and C. Importantly, the change patterns in stroke patients in microstate C relative to healthy controls were opposite to those in microstates A and B. Microstate integration and transmission were disrupted to some degree after stroke. These findings indicated the compensation and reorganization of neural dynamics after the disruption of neural function due to stroke. Moreover, we found that certain dynamic parameters were associated with lower limb function and spontaneous microstate dynamics seemed to encode information about motor ability. Overall, we extend our understanding of the brain dynamics, uncover the connectivity patterns underlying microstates, and provide new insights not obtained in studies using static features. Since the microstate dynamics can be modulated by neurofeedback and external stimulation (Michel and Koenig, 2018; Gui et al., 2020), our results open avenues for understanding the network reorganization and the development of new treatments for stroke patients.

## Data Availability Statement

The data supporting the findings of the present study (e.g., EEG features, microstate templates) are provided in Supplementary Material. In addition, preprocessed EEG recordings of the LEMON dataset are available for use at http://fcon_1000.projects.nitrc.org/indi/retro/MPI_LEMON.html.

## Ethics Statement

The studies involving human participants were reviewed and approved by the Ethics Committee of Beijing Tsinghua Changgung Hospital. The patients/participants provided their written informed consent to participate in this study.

## Author Contributions

ZH: conceptualization, data curation, methodology, software, formal analysis, validation, visualization, writing—original draft, and writing—review and editing. XZ and DC: data curation and writing—review and editing. YP: funding acquisition, project administration, and writing—review and editing. WD: funding acquisition, project administration, conceptualization, supervision, and writing—review and editing. All authors contributed to the article and approved the submitted version.

## Conflict of Interest

The authors declare that the research was conducted in the absence of any commercial or financial relationships that could be construed as a potential conflict of interest.

## Publisher’s Note

All claims expressed in this article are solely those of the authors and do not necessarily represent those of their affiliated organizations, or those of the publisher, the editors and the reviewers. Any product that may be evaluated in this article, or claim that may be made by its manufacturer, is not guaranteed or endorsed by the publisher.

## Footnotes

1
https://www.thomaskoenig.ch/index.php/software/microstates-in-eeglab/getting-started

2
https://github.com/okomarov/schemaball
