# Supplementary material for: EEG Microstate-Specific Functional Connectivity and Stroke-Related Alterations in Brain Dynamics
Source: Front Neurosci. 2022 May 11;16:848737. doi: 10.3389/fnins.2022.848737 (PMC9131012; doi:10.3389/fnins.2022.848737)
Supplement: Supplementary file 1 [file Data_Sheet_1.ZIP › Data Support the Fingings/Readme.docx]

The data that support the findings of the present study (e.g., EEG features, microstate templates) are provided. However, due to regulations, we are not able to share the EEG files of stroke patients and controls. In addition, preprocessed EEG recordings of the LEMON dataset were made available for use at <http://fcon_1000.projects.nitrc.org/indi/retro/MPI_LEMON.html>.

The accompanying .csv file " Raw Microstate Parameters of Patients and Controls" contains microstate parameters reported in the manuscript.

The “Template” folder contains the data files of the microstate templates and channel locations.

The “Microstate-wise FC” folder contains the data files of the microstate-wise connectivity for each group.
