# Supplementary material for: EEG Microstate-Specific Functional Connectivity and Stroke-Related Alterations in Brain Dynamics
Source: Front Neurosci. 2022 May 11;16:848737. doi: 10.3389/fnins.2022.848737 (PMC9131012; doi:10.3389/fnins.2022.848737)
Supplement: Supplementary file 2 [file Data_Sheet_2.pdf]

Supplementary Material

1 Supplementary Tables

Supplementary Table 1. Sample demographics of the LEMON dataset used in this study.

Filename	Gender	Age	Handedness
sub-032303	female	65–70	right
sub-032305	female	65–70	ambidexterity
sub-032308	female	60–65	right
sub-032318	male	60–65	right
sub-032329	female	65–70	right
sub-032331	male	70–75	right
sub-032333	male	70–75	left
sub-032337	female	65–70	right
sub-032338	male	65–70	right
sub-032340	female	65–70	left
sub-032347	male	75–80	right
sub-032369	male	60–65	right
sub-032375	male	65–70	right
sub-032377	female	65–70	right
sub-032378	male	55–60	right
sub-032391	male	35–40	right
sub-032392	female	65–70	right
sub-032395	female	60–65	right
sub-032429	male	30–35	right
sub-032444	male	30–35	right
sub-032447	male	60–65	right
sub-032450	female	60–65	right
sub-032454	male	60–65	right
sub-032457	female	70–75	right
sub-032462	male	65–70	right
sub-032475	male	65–70	right
sub-032490	male	70–75	right
sub-032491	male	70–75	right
sub-032494	male	65–70	right
sub-032495	male	70–75	right
sub-032496	male	60–65	right
sub-032497	male	60–65	right

Supplementary Table 2. Statistics of EEG preprocessing metrics for patients and controls.

Parameters	Patients_T0	Patients_T1	Controls	*p*	Effect size
**Length of raw EEG (seconds)**	258.7 ± 60.8<br>184.0–327.0	261.9 ± 60.9<br>184.0–322.0	228.5 ± 54.7<br>184.0–315.0	0.10	0.05
**Length of EEG retained (seconds)**	238.9 ± 54.6<br>175.1–305.6	243.1 ± 56.7<br>153.6–317.8	213.3 ± 49.8<br>162.2–301.4	0.11	0.05
**Removed channels (n)**	1.2 ± 1.3<br>0–4	1.2 ± 1.6<br>0–6	0.8 ± 1.3<br>0–5	0.52	0.02
**Rejected components (n)**	14.2 ± 6.6<br>4–28	13.7 ± 6.8<br>3–26	10.7 ± 4.2<br>4–21	0.15	0.02

Mean ± standard deviation and range for each parameter are provided in the table.
One-way ANOVA was performed across stroke patients at T0 and T1 and healthy controls of the length of raw EEG, length of EEG retained and removed channels; independent-samples Kruskal-Wallis test was performed for rejected components owning to violating the assumption of homogeneity of variance.
The eta-squared measure (η^2) is used to indicate the effect size.

Supplementary Table 3. Microstate-wise FC differences between each microstate pair for each group.

	Group pairs	B vs. A	C vs. A	C vs. B	D vs. A	D vs. B	D vs. C	E vs. A	E vs. B	E vs. C	E vs. D
**X vs. Y<br>X >Y**	**Controls**	**0.001**	**0.001**	**0.001**	**0.001**	**0.001**	**0.047<br>0.048**	**0.001**	**0.001**	**0.021**	**0.001**
	**LEMON**	**0.001**	**0.001**	**0.001**	**0.001**	**0.001**	**0.004**	**0.001**	**0.001**	**0.002**	**0.001**
	**Patients_T0**	**0.001**	**0.001**	**0.001**	**0.001**	**0.001**	**0.014**	**0.001**	**0.001**	**0.008**	**0.001**
	**Patients_T1**	**0.001**	**0.001**	**0.001**	**0.001**	**0.001**	**0.009**	**0.001**	**0.001**	**0.004**	**0.001**
**X vs. Y<br>X < Y**	**Controls**	**0.001**	0.096	**0.048<br>0.038**	**0.002**	**0.002**	**0.001**	**0.001**	**0.002**	**0.001**	**0.001**
	**LEMON**	**0.001**	**0.004**	**0.002**	**0.001**	**0.001**	**0.001**	**0.001**	**0.001**	**0.001**	**0.001**
	**Patients_T0**	**0.001**	**0.026**	**0.027**	**0.001**	**0.002**	**0.001**	**0.001**	**0.001**	**0.001**	**0.001**
	**Patients_T1**	**0.001**	**0.049**	0.099	**0.001**	**0.002**	**0.001**	**0.001**	**0.001**	**0.001**	**0.001**

p-values of the significant connected components are reported in the table.
For each X vs. Y, 2000 times permutations were performed.
Significant differences are indicated in bold.

Supplementary Table 4. Group average statistics (± SD) of the Patients at T0, Patients at T1, and Healthy Controls for all classes of GEV, mean duration, occurrence, coverage, mean interval, and mean GFP.

Parameter	Microstate	Patients at T0		Patients at T1		Controls	
		Mean	SD	Mean	SD	Mean	SD
GEV (%)	Class A	14.256	4.072	13.235	4.923	9.237	3.542
	Class B	12.294	4.422	11.750	4.971	8.170	2.568
	Class C	20.048	6.542	21.306	8.124	27.739	9.091
	Class D	4.837	2.272	5.218	1.855	5.057	2.108
	Class E	8.043	3.619	8.344	3.222	7.794	3.258
Mean Duration (ms)	Class A	61.948	4.446	61.265	5.529	57.570	4.226
	Class B	59.852	4.901	59.606	5.135	57.378	3.838
	Class C	68.632	9.981	71.039	12.342	82.243	18.594
	Class D	51.423	4.276	52.035	3.957	52.809	4.243
	Class E	54.629	4.540	55.352	4.906	56.548	5.125
Occurrence (Hz)	Class A	3.855	0.471	3.680	0.508	3.241	0.651
	Class B	3.559	0.595	3.420	0.672	3.070	0.564
	Class C	3.839	0.443	3.841	0.523	4.093	0.464
	Class D	2.355	0.635	2.466	0.494	2.513	0.612
	Class E	2.826	0.561	2.866	0.595	2.804	0.655
Coverage (%)	Class A	23.968	3.975	22.703	4.609	18.835	4.786
	Class B	21.516	4.983	20.650	5.378	17.739	3.947
	Class C	26.571	6.142	27.671	7.651	33.978	9.928
	Class D	12.301	3.970	12.935	3.163	13.393	3.947
	Class E	15.635	4.299	16.047	4.386	16.062	4.626
Mean Interval (ms)	Class A	202.032	37.650	216.871	50.796	265.043	75.438
	Class B	230.226	57.530	246.226	75.661	281.304	73.521
	Class C	195.355	35.094	194.645	46.566	165.591	39.040
	Class D	421.935	213.009	371.710	101.181	371.522	122.792
	Class E	313.032	78.218	310.774	90.554	323.261	115.269
Mean GFP (a.u.)	Class A	0.921	0.048	0.910	0.065	0.877	0.047
	Class B	0.907	0.049	0.903	0.054	0.866	0.047
	Class C	0.979	0.045	0.980	0.056	1.019	0.034
	Class D	0.819	0.071	0.827	0.065	0.826	0.064
	Class E	0.874	0.038	0.877	0.042	0.875	0.044

SD: standard deviation.

Supplementary Table 5. Patients at T0 vs. Controls for each microstate class of GEV, mean duration, occurrence, coverage, mean interval, and mean GFP.

Parameter	Microstate	p	p_{perm}	p_{holm}	$p_{perm\text{-}holm}$	Cohen's d
GEV	Class A	**<0.001**	**<0.001**	**<0.001**	**<0.001**	**1.301**
	Class B	**<0.001**	**<0.001**	**0.001**	**0.001**	**1.099**
	Class C	**0.001**	**0.001**	**0.002**	**0.002**	**−0.996**
	Class D	0.718	0.720	1.000	1.000	−0.100
	Class E	0.795	0.799	0.795	0.799	0.072
Mean Duration	Class A	**0.001**	**0.001**	**0.003**	**0.003**	**1.006**
	Class B	0.050	0.049	0.150	0.148	0.552
	Class C	**0.001**	**0.001**	**0.004**	**0.003**	**−0.954**
	Class D	0.243	0.243	0.243	0.243	−0.325
	Class E	0.152	0.153	0.304	0.306	−0.400
Occurrence	Class A	**<0.001**	**<0.001**	**0.001**	**0.001**	**1.108**
	Class B	**0.004**	**0.004**	**0.014**	**0.015**	**0.839**
	Class C	0.047	0.047	0.140	0.140	−0.561
	Class D	0.364	0.362	0.727	0.724	−0.252
	Class E	0.894	0.894	0.894	0.894	0.037
Coverage	Class A	**<0.001**	**<0.001**	**<0.001**	**<0.001**	**1.184**
	Class B	**0.004**	**0.004**	**0.012**	**0.012**	**0.826**
	Class C	**0.001**	**0.001**	**0.006**	**0.005**	**−0.930**
	Class D	0.321	0.320	0.642	0.640	−0.276
	Class E	0.728	0.731	0.728	0.731	−0.096
Mean Interval	Class A	**<0.001**	**<0.001**	**0.001**	**0.001**	**−1.109**
	Class B	**0.006**	**0.006**	**0.018**	**0.018**	**−0.789**
	Class C	**0.005**	**0.005**	**0.020**	**0.021**	**0.808**
	Class D	0.315	0.331	0.629	0.662	0.279
	Class E	0.699	0.707	0.699	0.707	−0.107
Mean GFP	Class A	**0.001**	**0.001**	**0.006**	**0.001**	**0.925**
	Class B	**0.003**	**0.003**	**0.010**	**0.003**	**0.849**
	Class C	**0.001**	**0.001**	**0.005**	**0.001**	**−0.964**
	Class D	0.704	0.708	1.000	0.708	−0.105
	Class E	0.927	0.926	0.927	0.926	−0.025

Post hoc pairwise group comparisons of microstate parameters between Patients at T0 and Controls. p values refer to two-sided independent sample t-tests and p_{perm} values refer to the permutation-based method (100000 times permutations). p_{holm} and $p_{perm\text{-}holm}$ values refer to Bonferroni-Holm corrected p-values for p and p_{perm} respectively. Cohen's d refers to two-sided independent sample t-tests. Significant differences survived correction are indicated in bold.

Supplementary Table 6. Patients at T1 vs. Controls for each microstate class of GEV, mean duration, occurrence, coverage, mean interval, and mean GFP.

Parameter	Microstate	p	p_{perm}	p_{holm}	$p_{perm\text{-}holm}$	Cohen's d
GEV	Class A	**0.002**	**0.001**	**0.009**	**0.006**	**0.910**
	Class B	**0.003**	**0.003**	**0.011**	**0.010**	**0.867**
	Class C	**0.009**	**0.009**	**0.026**	**0.027**	**−0.753**
	Class D	0.768	0.768	0.768	0.768	0.082
	Class E	0.540	0.538	1.000	1.000	0.170
Mean Duration	Class A	**0.010**	**0.009**	**0.050**	**0.045**	**0.736**
	Class B	0.086	0.085	0.259	0.256	0.481
	Class C	**0.010**	**0.010**	**0.041**	**0.040**	**−0.732**
	Class D	0.494	0.492	0.494	0.492	−0.189
	Class E	0.389	0.389	0.777	0.779	−0.239
Occurrence	Class A	**0.007**	**0.007**	**0.037**	**0.037**	**0.767**
	Class B	0.049	0.048	0.194	0.191	0.556
	Class C	0.072	0.072	0.217	0.217	−0.505
	Class D	0.758	0.756	0.758	0.756	−0.085
	Class E	0.717	0.716	1.000	1.000	0.100
Coverage	Class A	**0.004**	**0.005**	**0.021**	**0.023**	**0.826**
	Class B	0.033	0.034	0.099	0.102	0.603
	Class C	**0.011**	**0.011**	**0.044**	**0.043**	**−0.726**
	Class D	0.637	0.637	1.000	1.000	−0.131
	Class E	0.991	0.991	0.991	0.991	−0.003
Mean Interval	Class A	**0.007**	**0.006**	**0.035**	**0.031**	**−0.772**
	Class B	0.094	0.094	0.283	0.281	−0.469
	Class C	0.019	0.019	0.075	0.076	0.667
	Class D	0.995	0.996	0.995	0.996	0.002
	Class E	0.657	0.661	1.000	1.000	−0.123
Mean GFP	Class A	0.040	0.039	0.120	0.116	0.579
	Class B	**0.012**	**0.012**	**0.048**	**0.049**	**0.716**
	Class C	**0.005**	**0.003**	**0.023**	**0.015**	**−0.814**
	Class D	0.973	0.972	0.973	0.972	0.009
	Class E	0.874	0.875	1.000	1.000	0.044

Post hoc pairwise group comparisons of microstate parameters between Patients at T1 and Controls. p values refer to two-sided independent sample t-tests and p_{perm} values refer to the permutation-based method (100000 times permutations). p_{holm} and $p_{perm\text{-}holm}$ values refer to Bonferroni-Holm corrected p-values for p and p_{perm} respectively. Cohen's d refers to two-sided independent sample t-tests. Significant differences survived correction are indicated in bold.

Supplementary Table 7. Patients vs. Controls for Spatial Correlation metric (SC) and Transition Probability (TP).

Timepoint	Parameters	p	p_{perm}	p_{holm}	$p_{perm\text{-}holm}$	Cohen's d
T0	SC_{AA}	**0.001**	**0.001**	**0.031**	**0.032**	**0.938**
	SC_{AB}	**0.001**	**0.002**	**0.031**	**0.037**	**0.934**
	SC_{BA}	**0.001**	**0.001**	**0.028**	**0.032**	**0.950**
	SC_{BB}	**0.002**	**0.002**	**0.034**	**0.040**	**0.919**
	SC_{EE}	0.049	0.048	1.000	1.000	0.554
	TP_{BA}	0.030	0.030	0.504	0.513	0.616
	TP_{BC}	0.038	0.037	0.604	0.588	−0.587
	TP_{DC}	0.024	0.022	0.427	0.403	−0.641
	TP_{EA}	0.004	0.004	0.074	0.071	0.836
	TP_{EC}	0.007	0.005	0.124	0.089	−0.780
T1	SC_{AA}	0.003	0.003	0.059	0.058	0.868
	SC_{AB}	**0.001**	**0.001**	**0.028**	**0.030**	**0.951**
	SC_{BA}	**0.001**	**0.001**	**0.031**	**0.032**	**0.936**
	SC_{BB}	**0.002**	**0.002**	**0.046**	**0.050**	**0.895**
	SC_{DD}	0.018	0.018	0.379	0.378	0.672
	SC_{EE}	0.031	0.032	0.629	0.632	0.608
	TP_{EA}	0.007	0.007	0.143	0.135	0.771

Post hoc pairwise group comparisons of SC and TP for Patients at T0 vs. Controls and Patients at T1 vs. Controls. p values refer to two-sided independent sample t-tests and p_{perm} values refer to the permutation-based method (100000 times permutations). p_{holm} and $p_{perm\text{-}holm}$ values refer to Bonferroni-Holm corrected p-values for p and p_{perm} respectively. Cohen's d refers to two-sided independent sample t-tests. Significant differences survived correction are indicated in bold. Only the items their p_{perm} values < 0.05 are provided in this table.

Supplementary Table 8. Two-sided correlation analysis between microstate parameters (GEV, mean duration, occurrence, coverage, mean interval, and mean GFP) and FMAL.

Parameter	Microstate	Parameter_T0–FMAL_T0	Parameter_T1–FMAL_T1
GEV	Class A	***r* = −0.386, *p* = 0.032, CI [−0.575, −0.097]**	*r* = −0.107, *p* = 0.565, CI [−0.369, 0.295]
	Class B	*r* = −0.011, *p* = 0.953, CI [−0.298, 0.311]	*r* = 0.232, *p* = 0.210, CI [−0.100, 0.530]
	Class C	*r* = −0.066, *p* = 0.725, CI [−0.413, 0.239]	*r* = −0.305, *p* = 0.094, CI [−0.595, 0.061]
	Class D	*r* = 0.176, *p* = 0.345, CI [−0.130, 0.451]	*r* = 0.334, *p* = 0.067, CI [−0.059, 0.640]
	Class E	*r* = −0.091, *p* = 0.631, CI [−0.565, 0.287]	*r* = 0.052, *p* = 0.777, CI [−0.372, 0.428]
Mean Duration	Class A	*r* = −0.251, *p* = 0.173, CI [−0.482, 0.020]	*r* = −0.073, *p* = 0.696, CI [−0.359, 0.308]
	Class B	*r* = 0.046, *p* = 0.806, CI [−0.254, 0.383]	*r* = 0.318, *p* = 0.080, CI [−0.086, 0.610]
	Class C	*r* = −0.064, *p* = 0.735, CI [−0.364, 0.225]	*r* = −0.272, *p* = 0.138, CI [−0.582, 0.057]
	Class D	*r* = 0.201, *p* = 0.279, CI [−0.095, 0.506]	***r* = 0.497, *p* = 0.005, CI [0.185, 0.703]**
	Class E	*r* = −0.114, *p* = 0.540, CI [−0.544, 0.253]	*r* = 0.131, *p* = 0.484, CI [−0.192, 0.411]
Occurrence	Class A	*r* = −0.223, *p* = 0.228, CI [−0.481, 0.113]	*r* = −0.072, *p* = 0.700, CI [−0.355, 0.338]
	Class B	*r* = 0.111, *p* = 0.552, CI [−0.189, 0.421]	*r* = 0.240, *p* = 0.192, CI [−0.115, 0.606]
	Class C	*r* = −0.023, *p* = 0.904, CI [−0.386, 0.265]	***r* = −0.411, *p* = 0.021, CI [−0.649, −0.047]**
	Class D	*r* = 0.239, *p* = 0.196, CI [−0.101, 0.528]	***r* = 0.430, *p* = 0.016, CI [0.031, 0.715]**
	Class E	*r* = 0.102, *p* = 0.583, CI [−0.386, 0.481]	*r* = 0.039, *p* = 0.837, CI [−0.402, 0.468]
Coverage	Class A	*r* = −0.271, *p* = 0.140, CI [−0.516, 0.052]	*r* = −0.089, *p* = 0.632, CI [−0.376, 0.311]
	Class B	*r* = 0.094, *p* = 0.615, CI [−0.201, 0.400]	*r* = 0.274, *p* = 0.135, CI [−0.097, 0.618]
	Class C	*r* = −0.064, *p* = 0.732, CI [−0.408, 0.232]	***r* = −0.372, *p* = 0.040, CI [−0.650, −0.033]**
	Class D	*r* = 0.224, *p* = 0.225, CI [−0.093, 0.530]	***r* = 0.480, *p* = 0.006, CI [0.079, 0.743]**
	Class E	*r* = 0.025, *p* = 0.834, CI [−0.473, 0.413]	*r* = 0.059, *p* = 0.756, CI [−0.355, 0.449]
Mean Interval	Class A	*r* = 0.231, *p* = 0.213, CI [−0.080, 0.479]	*r* = 0.066, *p* = 0.725, CI [−0.346, 0.334]
	Class B	*r* = −0.123, *p* = 0.511, CI [−0.450, 0.166]	*r* = −0.271, *p* = 0.138, CI [−0.641, 0.081]
	Class C	*r* = 0.025, *p* = 0.895, CI [−0.266, 0.365]	***r* = 0.394, *p* = 0.027, CI [0.046, 0.645]**
	Class D	*r* = −0.282, *p* = 0.121, CI [−0.610, 0.004]	***r* = −0.512, *p* = 0.003, CI [−0.755, −0.045]**
	Class E	*r* = −0.072, *p* = 0.704, CI [−0.479, 0.415]	*r* = −0.133, *p* = 0.478, CI [−0.594, 0.283]
Mean GFP	Class A	*r* = −0.077, *p* = 0.683, CI [−0.363, 0.224]	*r* = 0.041, *p* = 0.827, CI [−0.262, 0.352]
	Class B	*r* = 0.179, *p* = 0.340, CI [−0.114, 0.460]	*r* = 0.215, *p* = 0.244, CI [−0.102, 0.466]
	Class C	*r* = 0.298, *p* = 0.104, CI [0.067, 0.502]	*r* = −0.056, *p* = 0.766, CI [−0.378, 0.254]
	Class D	***r* = 0.359, *p* = 0.046, CI [0.072, 0.627]**	*r* = 0.106, *p* = 0.568, CI [−0.243, 0.372]
	Class E	*r* = 0.081, *p* = 0.667, CI [−0.299, 0.442]	*r* = 0.133, *p* = 0.475, CI [−0.297, 0.483]

Significant correlations are indicated in bold. Pearson correlation coefficient (*r*) and the corresponding permutation-based *p*-value are given. 95% confidence interval was computed by the bootstrap method (bias corrected and accelerated) with 10000 bootstrap data samples. *p*-values are not corrected for multiple comparisons.

Supplementary Table 9. Two-sided correlation analysis between SC/TP and FMAL.

Microstate Parameter		Parameter_T0–FMAL_T0	Parameter_T1–FMAL_T1
Spatial Correlation metrics	SC_{AA}	**r = −0.420, p = 0.020, CI [−0.650, −0.087]**	r = −0.043, p = 0.822, CI [−0.356, 0.293]
	SC_{AB}	**r = −0.387, p = 0.032, CI [−0.620, −0.081]**	r = 0.064, p = 0.731, CI [−0.268, 0.384]
	SC_{AC}	**r = −0.401, p = 0.025, CI [−0.647, −0.068]**	r = −0.290, p = 0.111, CI [−0.573, 0.046]
	SC_{AD}	**r = −0.369, p = 0.040, CI [−0.631, 0.023]**	r = −0.223, p = 0.228, CI [−0.562, 0.246]
	SC_{AE}	**r = −0.379, p = 0.034, CI [−0.643, −0.062]**	r = −0.193, p = 0.298, CI [−0.521, 0.166]
	SC_{CA}	**r = −0.391, p = 0.029, CI [−0.633, −0.001]**	r = −0.305, p = 0.095, CI [−0.579, 0.090]
	SC_{EE}	**r = 0.363, p = 0.045, CI [−0.610, −0.020]**	r = −0.072, p = 0.701, CI [−0.430, 0.244]
Transision Probability	TP_{CA}	**r = −0.384, p = 0.033, CI [−0.609, −0.105]**	r = −0.275, p = 0.134, CI [−0.618, 0.173]

Significant correlations are indicated in bold. Pearson correlation coefficient (r) and the corresponding permutation-based p-value are given. 95% confidence interval was computed by the bootstrap method (bias corrected and accelerated) with 10000 bootstrap data samples. Only the items at T0 their p values < 0.05 are provided in this table. At T1, there was no significant result. p-values are not corrected for multiple comparisons.

2 Supplementary Figures

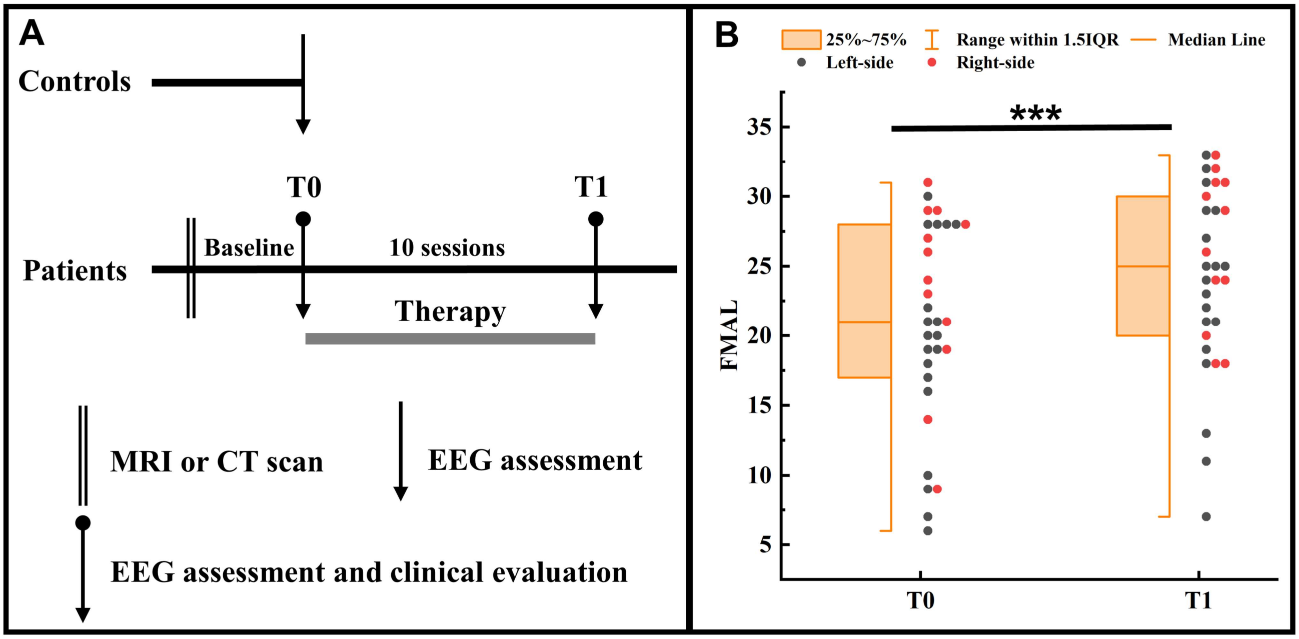

Supplementary Figure 1. Experimental setup. **(A)** Experiment timeline. Structural MRI or CT scans were acquired before the rehabilitation therapy. For stroke patients, EEG assessments and clinical evaluations were performed at baseline, then at end of 10 training sessions for all patients. EEG data were only acquired at baseline for healthy controls. **(B)** Stroke patients showed significant gains in lower-extremity motor (p < 0.001). Left-side: patients with left-sided stroke; Right-side: patients with right-sided stroke.

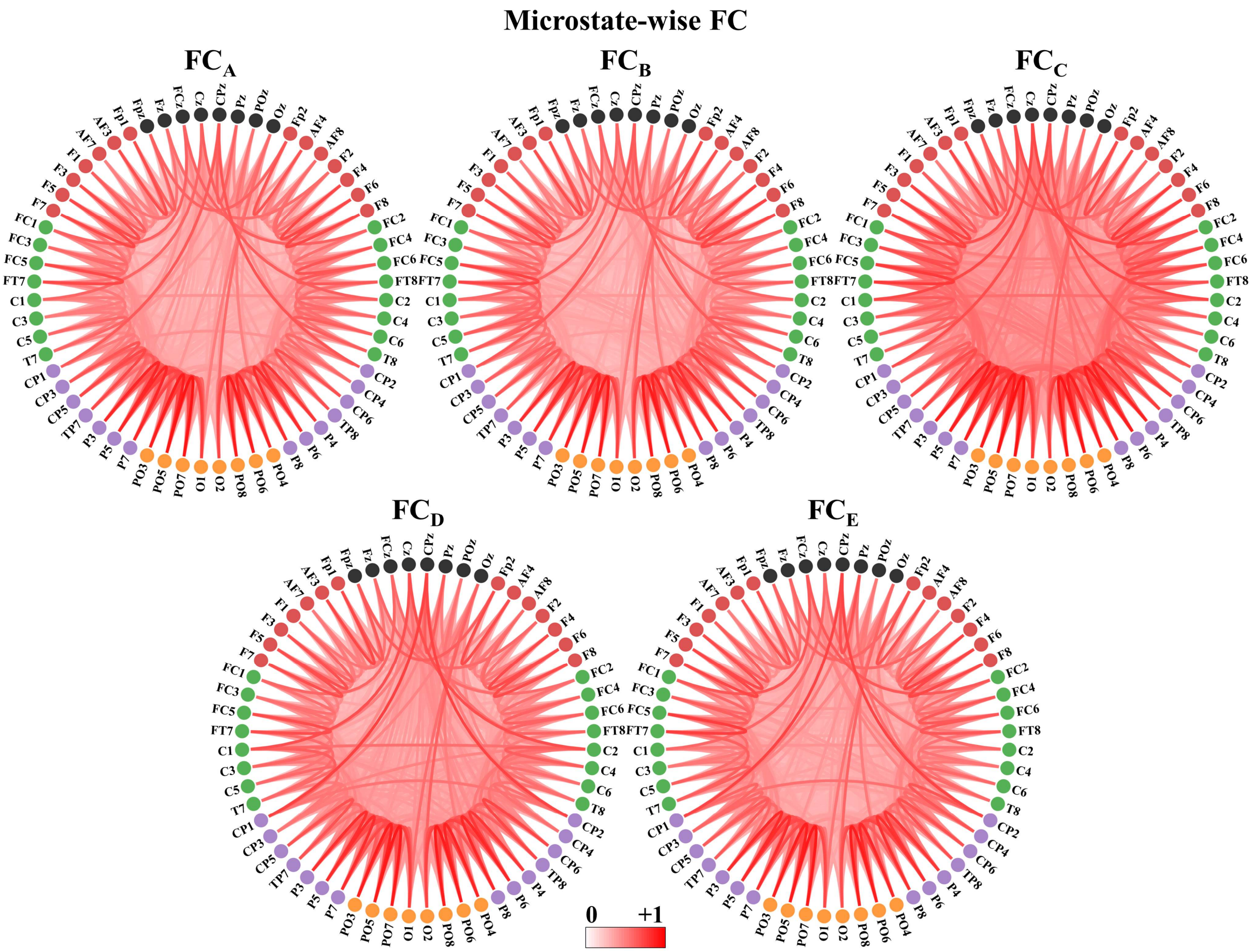

Supplementary Figure 2. Microstate-wise functional connectivity. Color depth indicates the strength of the functional connectivity. FC_X: functional connectivity of microstate X.

Microstate B vs. Microstate A (microstate-wise FC)

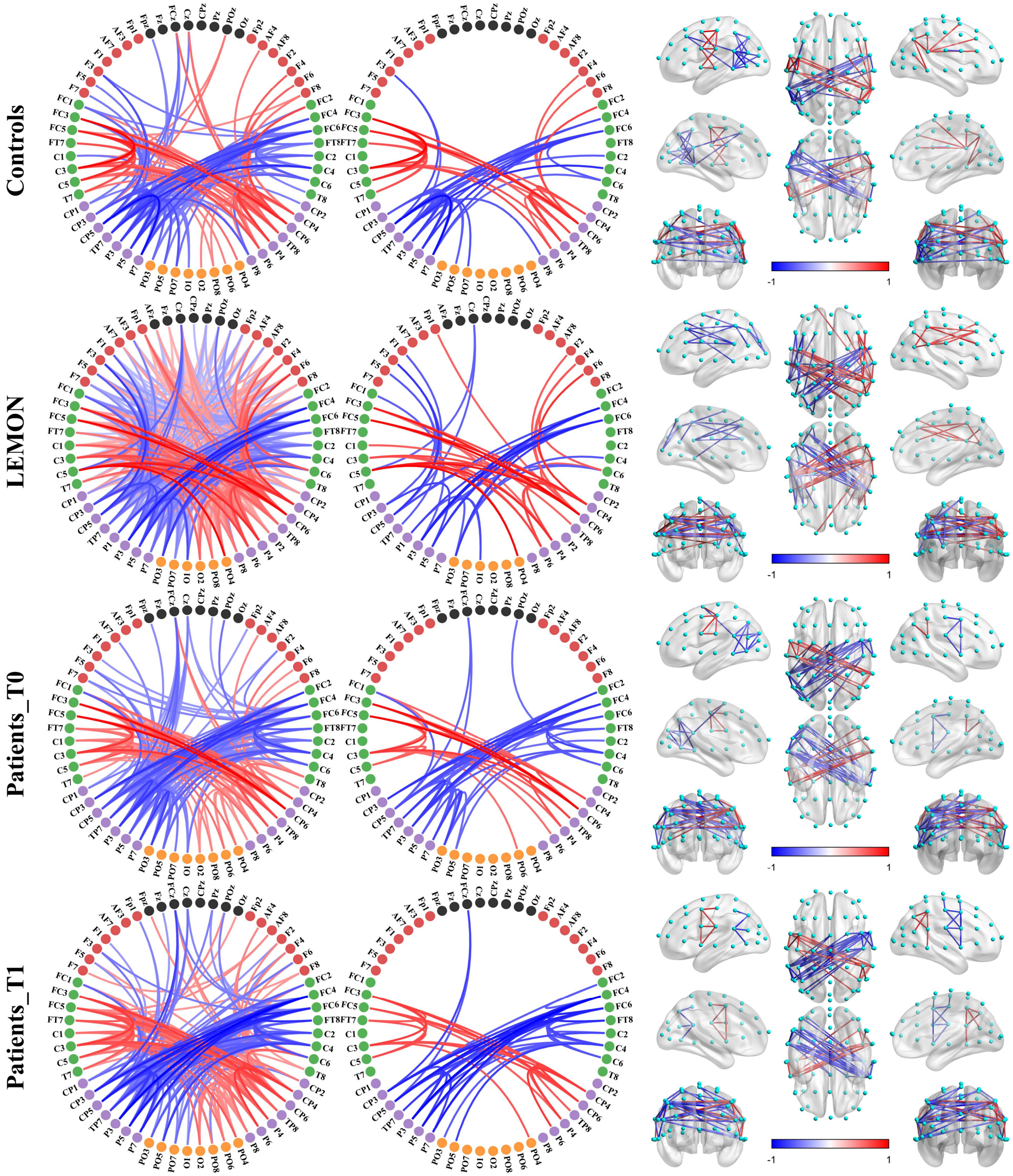

Supplementary Figure 3. Results of FC_B versus FC_A for four groups. The red color indicates greater connectivity for microstate B versus microstate A, while the blue color indicates the opposite pattern. Color depth indicates the size of the difference. Only the significant connected components are displayed. First column: original components; second and third columns: top 50 connections.

Microstate C vs. Microstate A (microstate-wise FC)

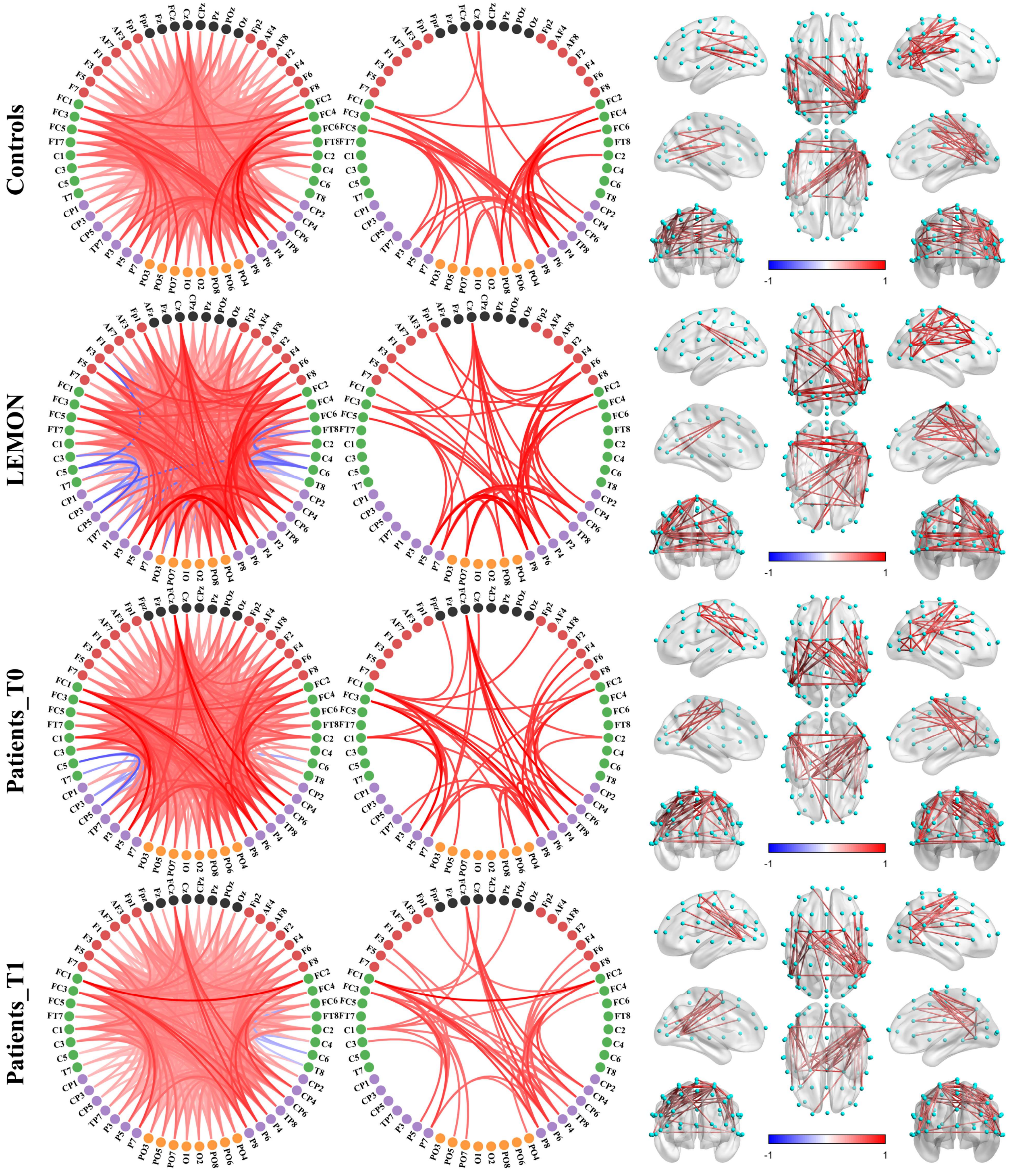

Supplementary Figure 4. Results of FC_C versus FC_A for four groups. The red color indicates greater connectivity for microstate C versus microstate A, while the blue color indicates the opposite pattern. Color depth indicates the size of the difference. Only the significant connected components are displayed. First column: original components; second and third columns: top 50 connections.

Microstate C vs. Microstate B (microstate-wise FC)

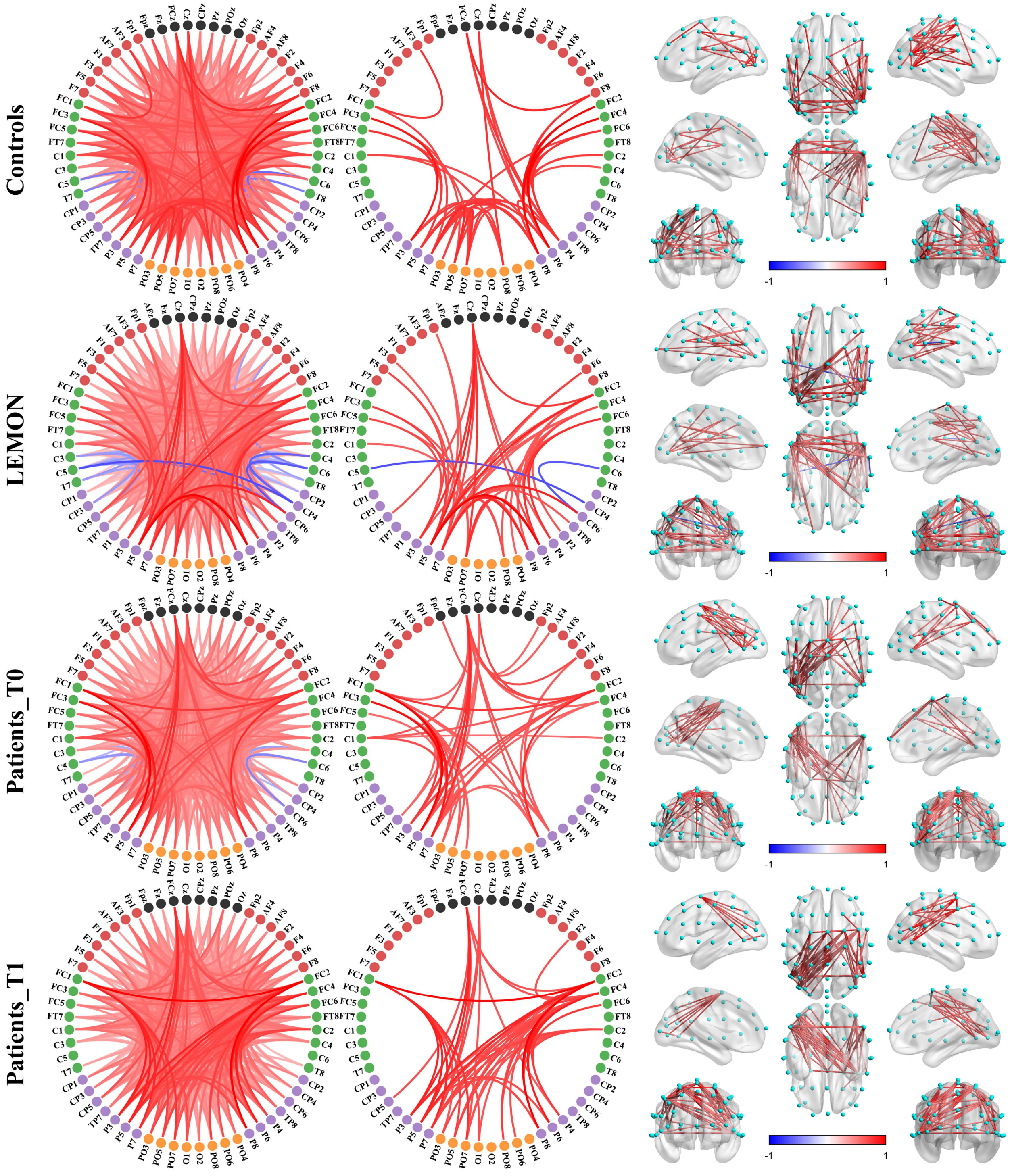

Supplementary Figure 5. Results of FC_C versus FC_B for four groups. The red color indicates greater connectivity for microstate C versus microstate B, while the blue color indicates the opposite pattern. Color depth indicates the size of the difference. Only the significant connected components are displayed. First column: original components; second and third columns: top 50 connections.

Microstate D vs. Microstate A (microstate-wise FC)

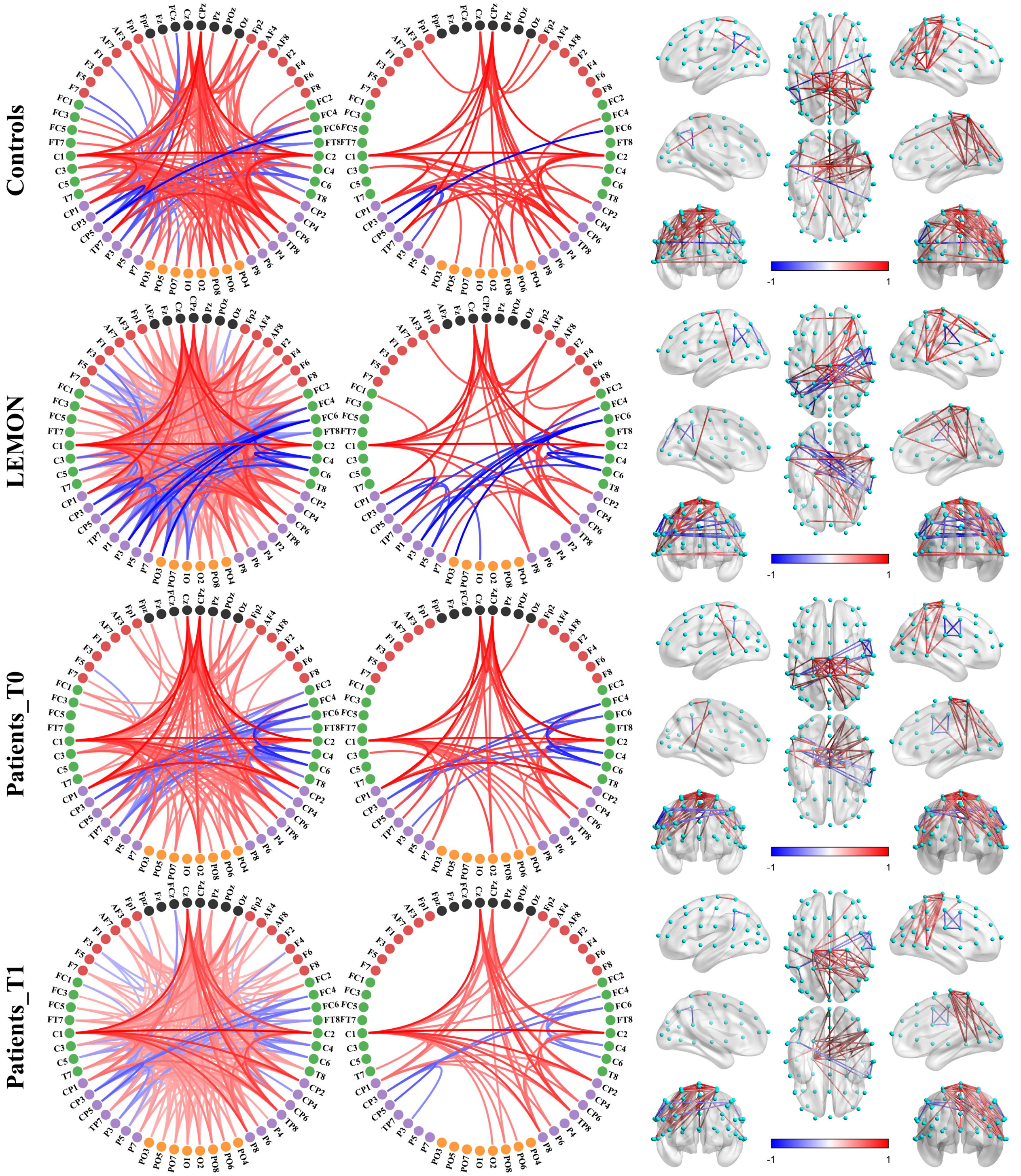

Supplementary Figure 6. Results of FC_D versus FC_A for four groups. The red color indicates greater connectivity for microstate D versus microstate A, while the blue color indicates the opposite pattern. Color depth indicates the size of the difference. Only the significant connected components are displayed. First column: original components; second and third columns: top 50 connections.

Microstate D vs. Microstate B (microstate-wise FC)

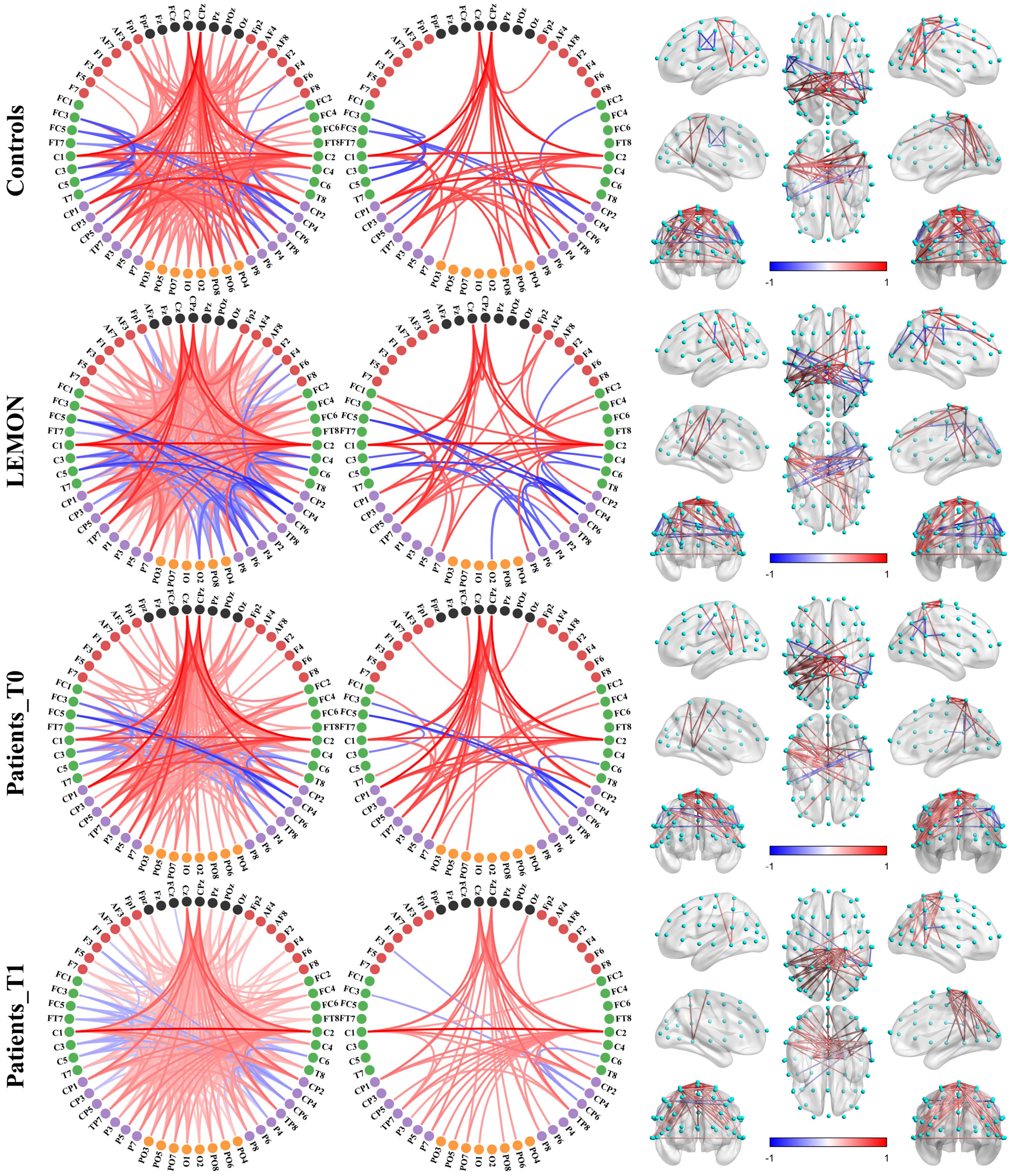

Supplementary Figure 7. Results of FC_D versus FC_B for four groups. The red color indicates greater connectivity for microstate D versus microstate B, while the blue color indicates the opposite pattern. Color depth indicates the size of the difference. Only the significant connected components are displayed. First column: original components; second and third columns: top 50 connections.

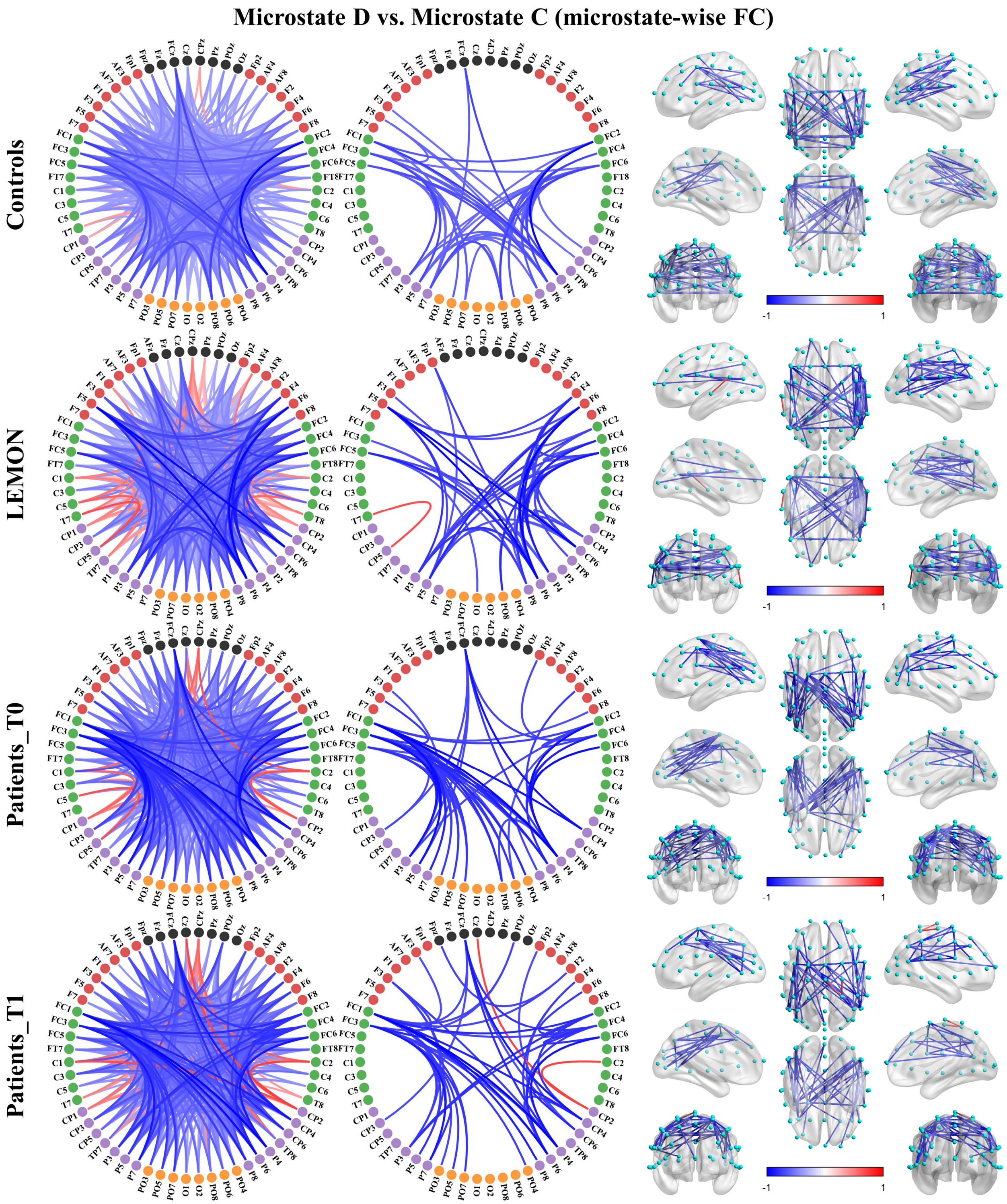

Supplementary Figure 8. Results of FC_D versus FC_C for four groups. The red color indicates greater connectivity for microstate D versus microstate C, while the blue color indicates the opposite pattern. Color depth indicates the size of the difference. Only the significant connected components are displayed. First column: original components; second and third columns: top 50 connections.

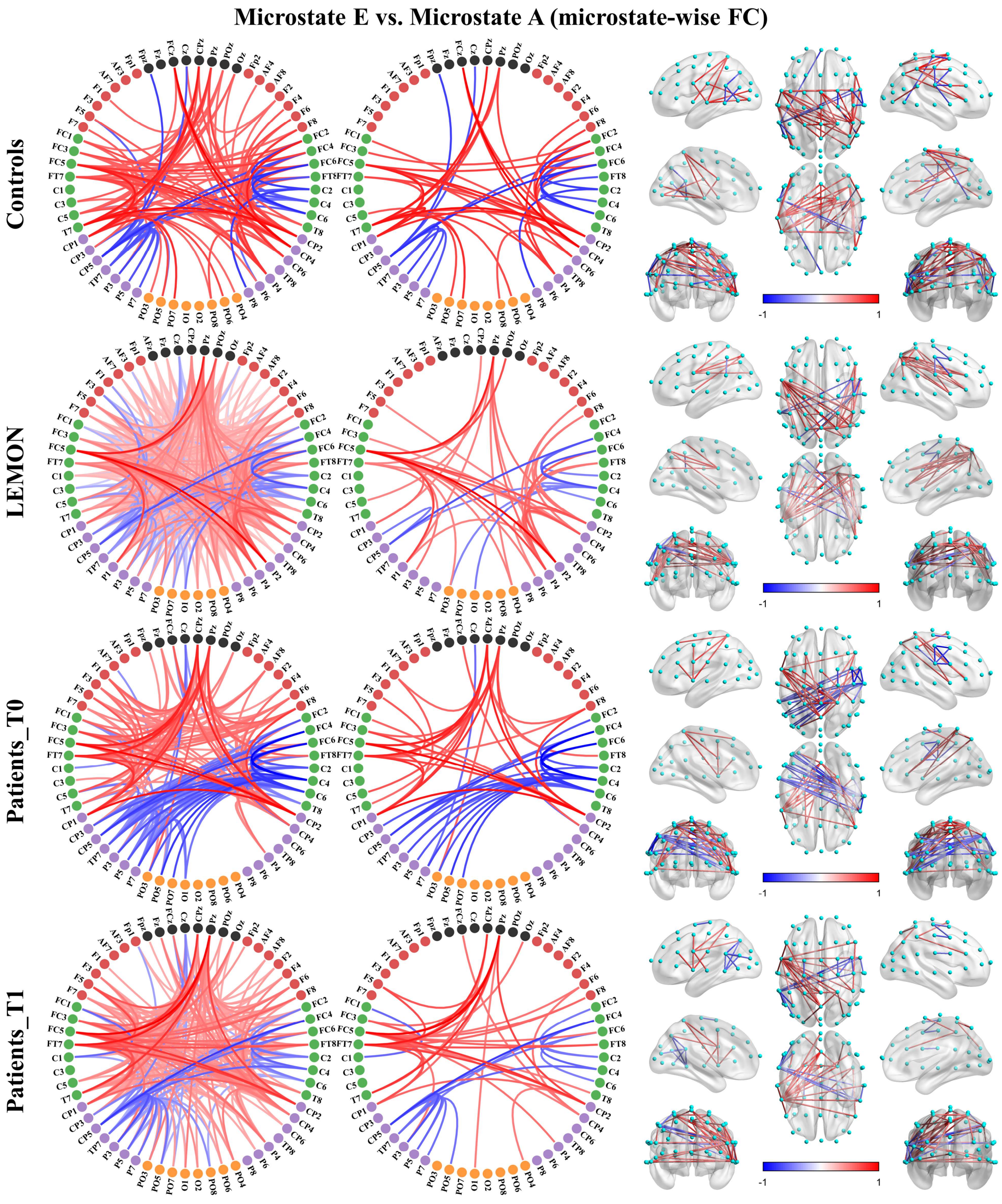

Supplementary Figure 9. Results of FC_E versus FC_A for four groups. The red color indicates greater connectivity for microstate E versus microstate A, while the blue color indicates the opposite pattern. Color depth indicates the size of the difference. Only the significant connected components are displayed. First column: original components; second and third columns: top 50 connections.

Microstate E vs. Microstate B (microstate-wise FC)

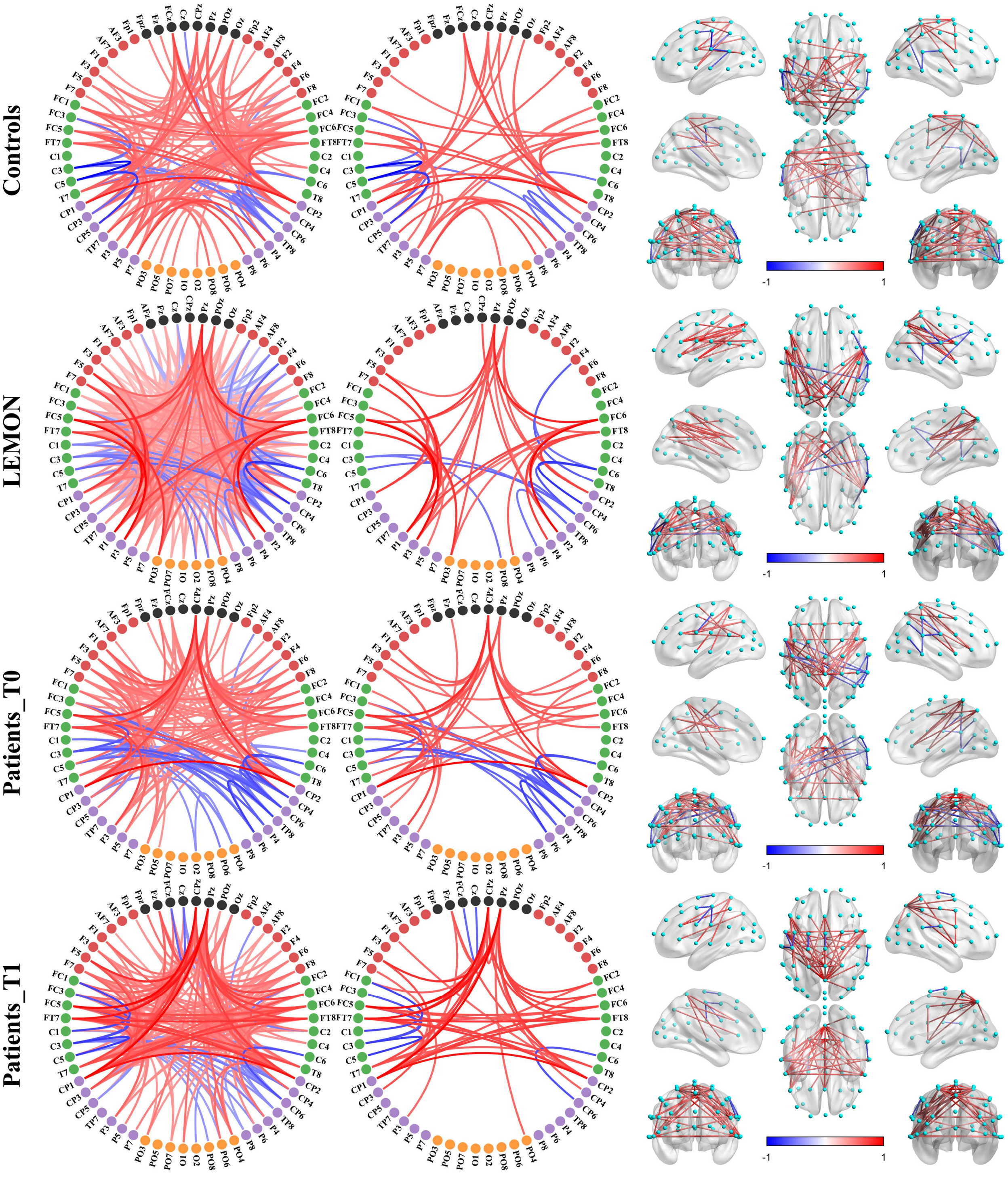

Supplementary Figure 10. Results of FC_E versus FC_B for four groups. The red color indicates greater connectivity for microstate E versus microstate B, while the blue color indicates the opposite pattern. Color depth indicates the size of the difference. Only the significant connected components are displayed. First column: original components; second and third columns: top 50 connections.

Microstate E vs. Microstate C (microstate-wise FC)

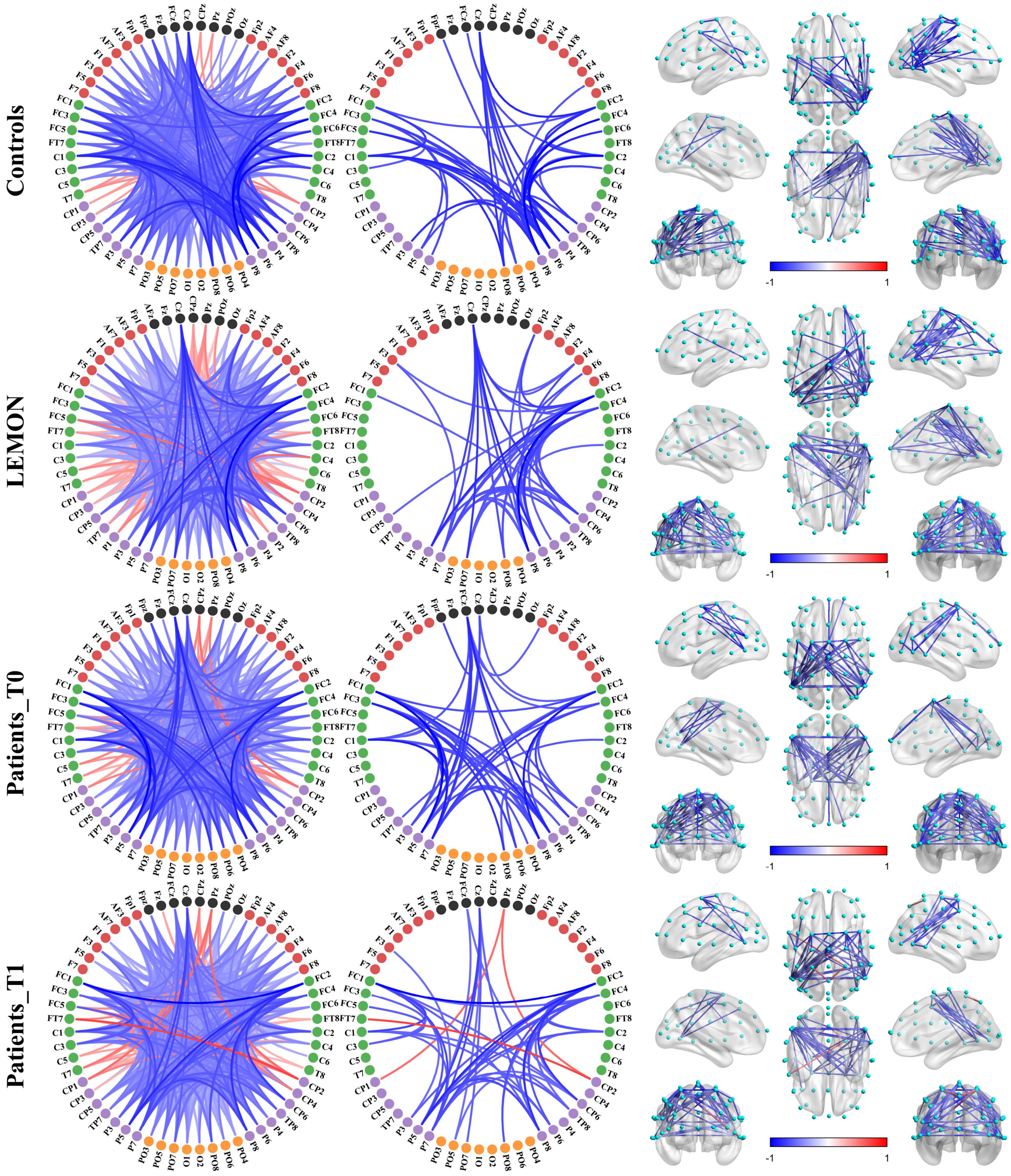

Supplementary Figure 11. Results of FC_E versus FC_C for four groups. The red color indicates greater connectivity for microstate E versus microstate C, while the blue color indicates the opposite pattern. Color depth indicates the size of the difference. Only the significant connected components are displayed. First column: original components; second and third columns: top 50 connections.

Microstate E vs. Microstate D (microstate-wise FC)

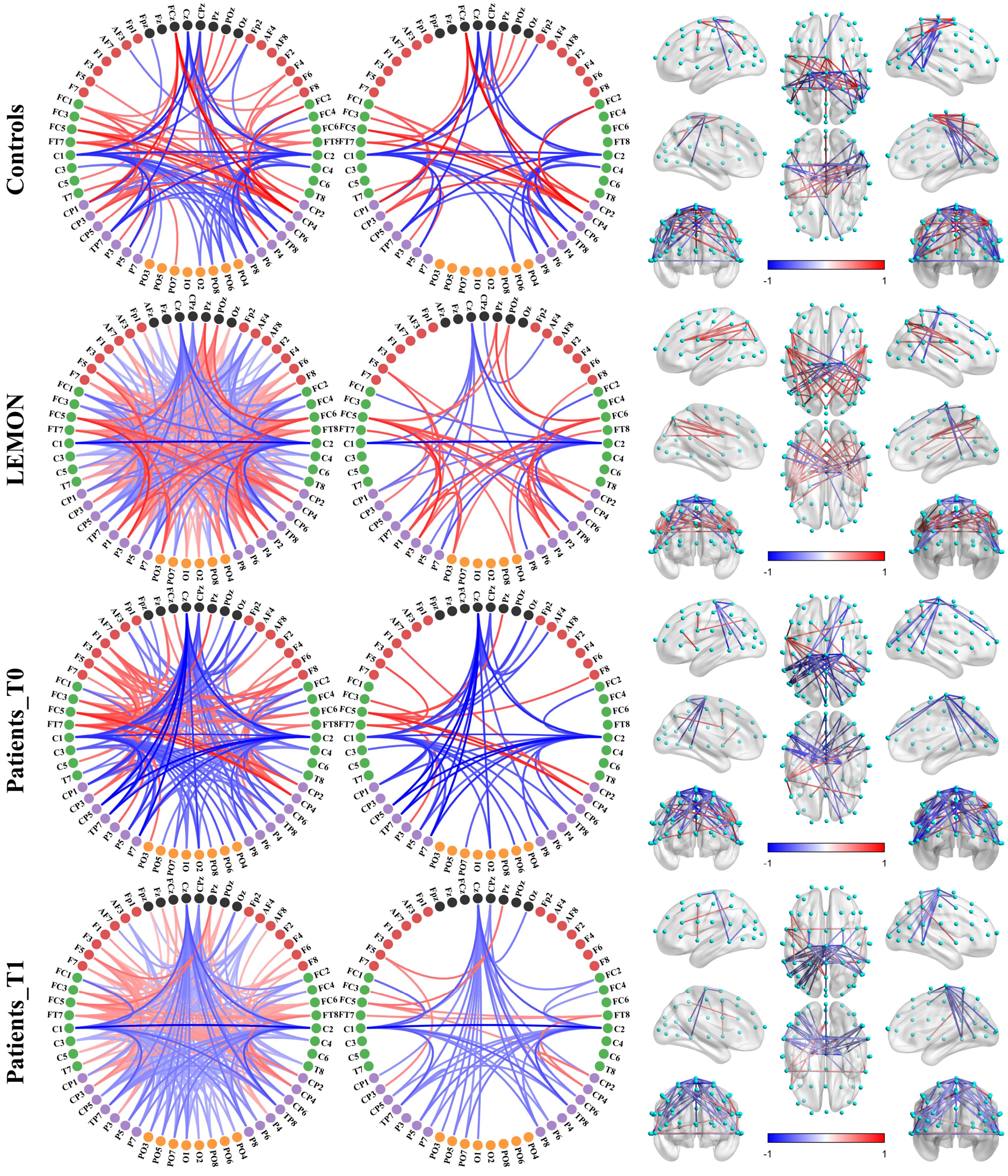

Supplementary Figure 12. Results of FC_E versus FC_D for four groups. The red color indicates greater connectivity for microstate E versus microstate D, while the blue color indicates the opposite pattern. Color depth indicates the size of the difference. Only the significant connected components are displayed. First column: original components; second and third columns: top 50 connections.

Patients_left_side_stroke_T0

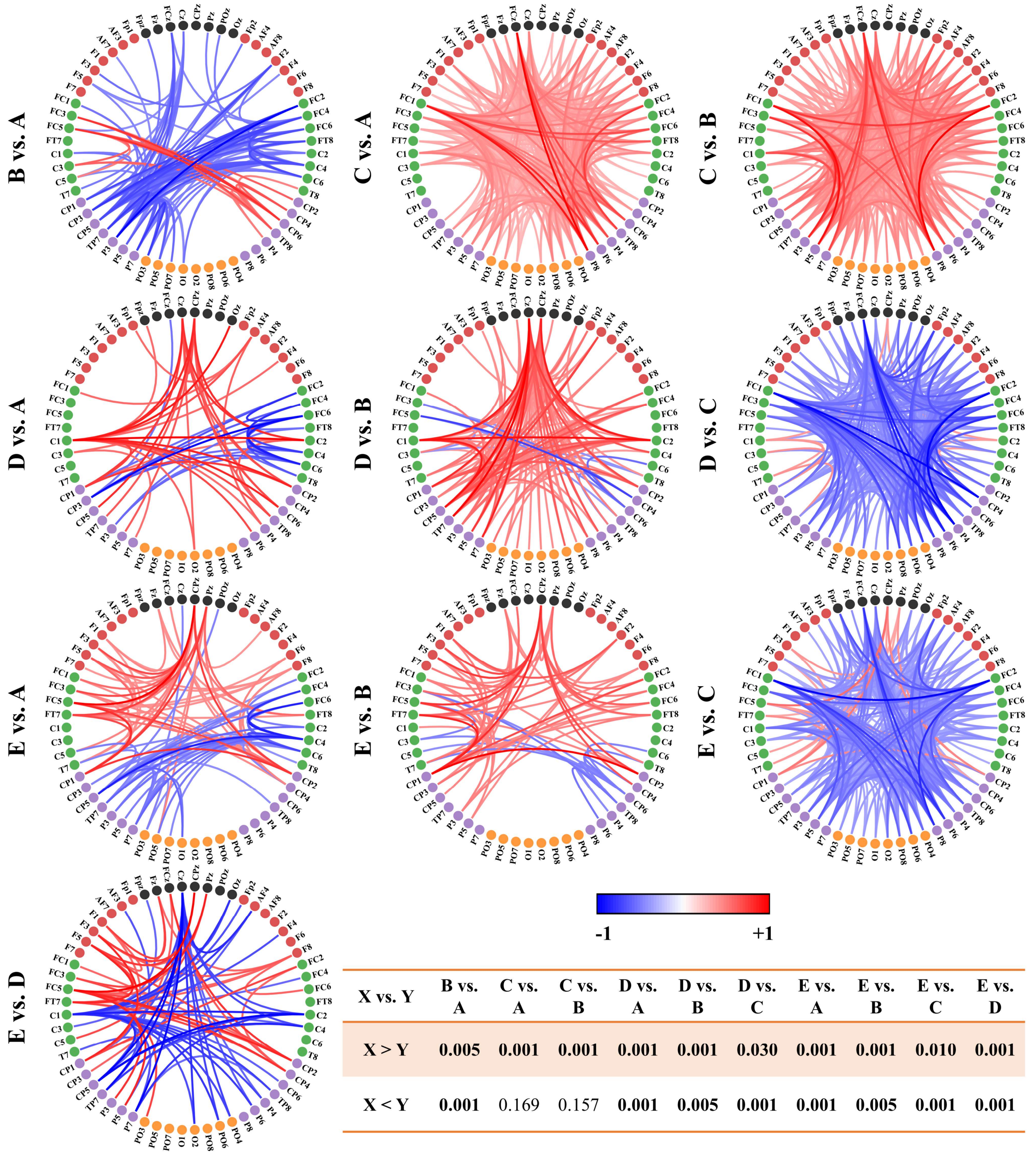

X vs. Y	B vs. A	C vs. A	C vs. B	D vs. A	D vs. B	D vs. C	E vs. A	E vs. B	E vs. C	E vs. D
X > Y	**0.005**	**0.001**	**0.001**	**0.001**	**0.001**	**0.030**	**0.001**	**0.001**	**0.010**	**0.001**
X < Y	**0.001**	0.169	0.157	**0.001**	**0.005**	**0.001**	**0.001**	**0.005**	**0.001**	**0.001**

Supplementary Figure 13. Results of microstate-wise functional connectivity comparisons for patients with left-sided stroke at T0. For X vs. Y, the red color indicates X > Y, while the blue color indicates the opposite pattern. Color depth indicates the size of the difference. Only the significant connected components are displayed. *p*-values are also provided in the figure.

Patients_left_side_stroke_T1

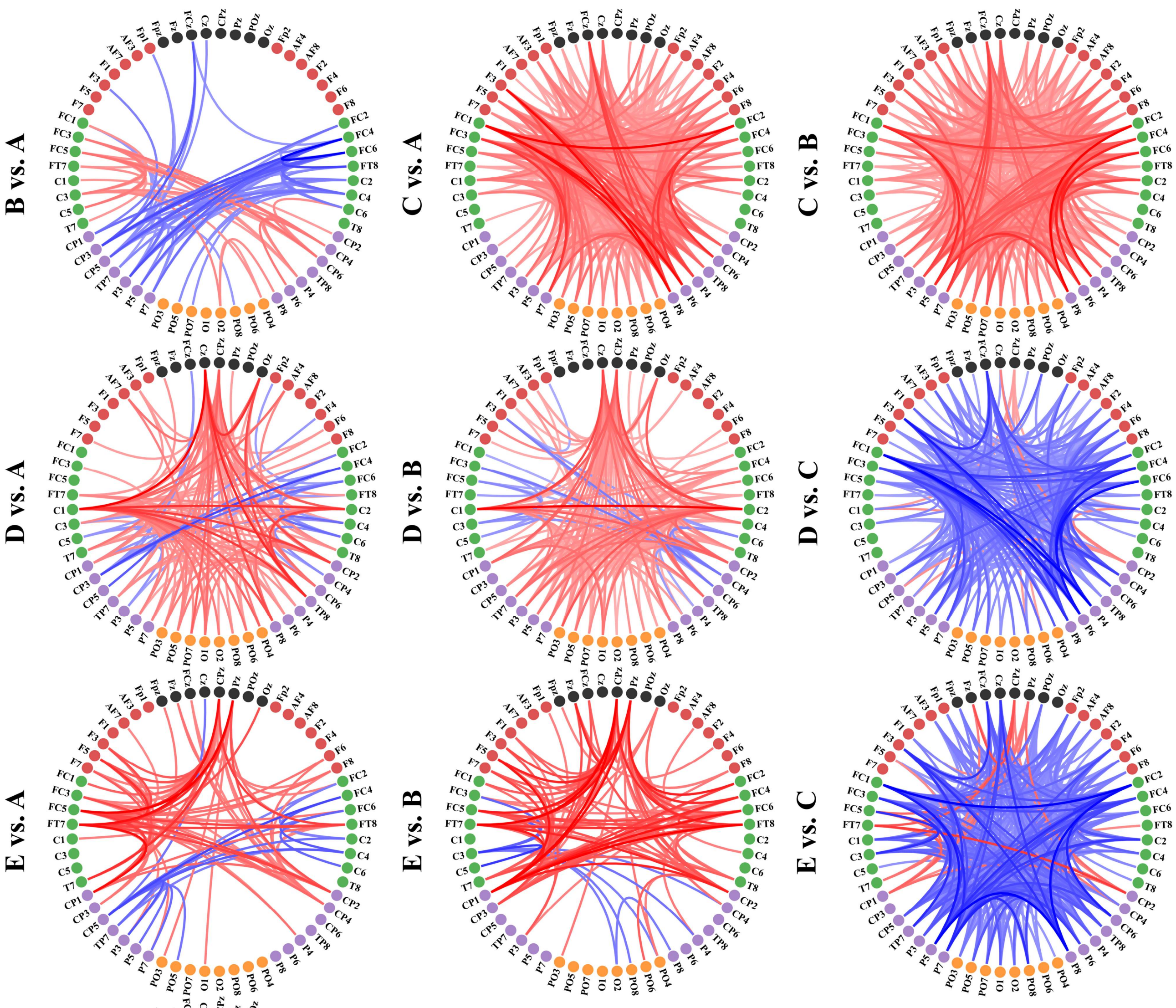

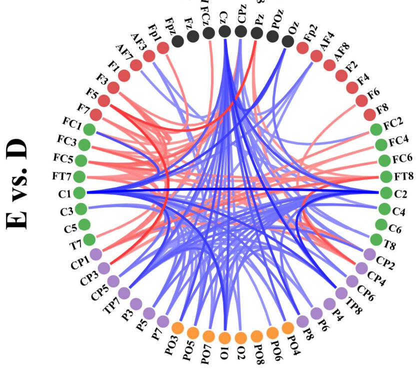

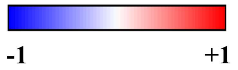

X vs. Y	B vs. A	C vs. A	C vs. B	D vs. A	D vs. B	D vs. C	E vs. A	E vs. B	E vs. C	E vs. D
X > Y	**0.002**	**0.001**	**0.001**	**0.001**	**0.001**	**0.015**	**0.001**	**0.001**	**0.005**	**0.001**
X < Y	**0.001**	0.229	None	**0.003**	**0.002**	**0.001**	**0.006**	**0.007**	**0.001**	**0.001**

Supplementary Figure 14. Results of microstate-wise functional connectivity comparisons for patients with left-sided stroke at T1. For X vs. Y, the red color indicates X > Y, while the blue color indicates the opposite pattern. Color depth indicates the size of the difference. Only the significant connected components are displayed. *p*-values are also provided in the figure.

Patients_right_side_stroke_T0

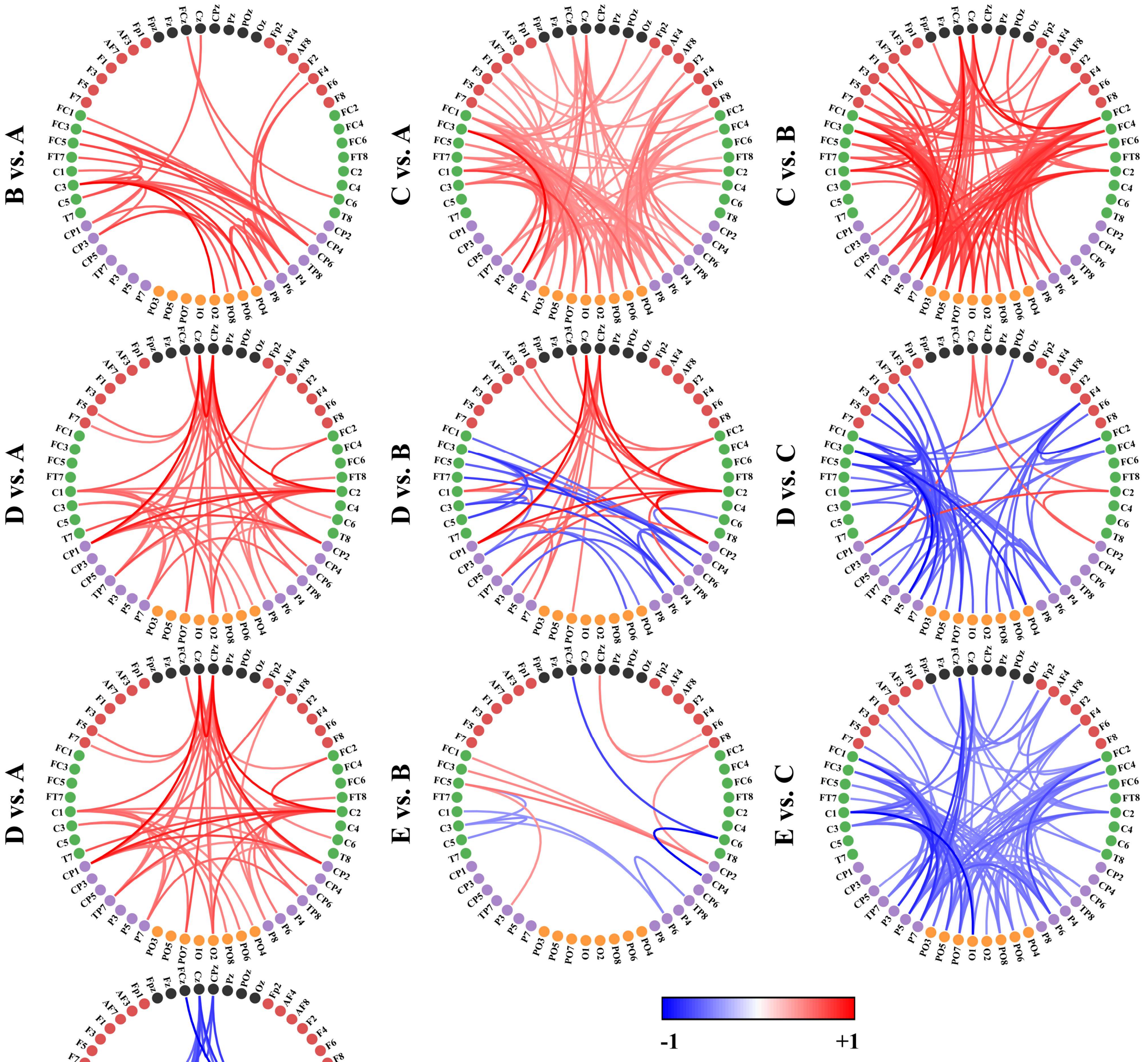

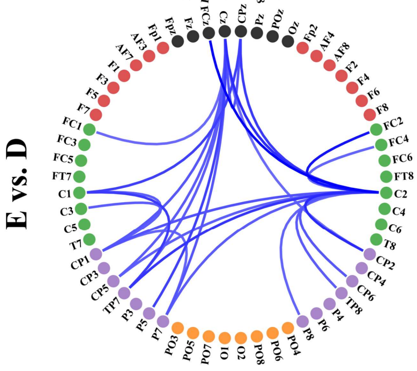

X vs. Y	B vs. A	C vs. A	C vs. B	D vs. A	D vs. B	D vs. C	E vs. A	E vs. B	E vs. C	E vs. D
**X > Y**	**0.001**	**0.001**	**0.001**	**0.001**	**0.001**	**0.024**	**0.001**	**0.007**	None	0.137
**X < Y**	0.465	None	0.060	0.061	**0.004**	**0.001**	**0.025**	**0.016**<br>**0.010**	**0.001**	**0.001**

Supplementary Figure 15. Results of microstate-wise functional connectivity comparisons for patients with right-sided stroke at T0. For X vs. Y, the red color indicates X > Y, while the blue color indicates the opposite pattern. Color depth indicates the size of the difference. Only the significant connected components are displayed. *p*-values are also provided in the figure.

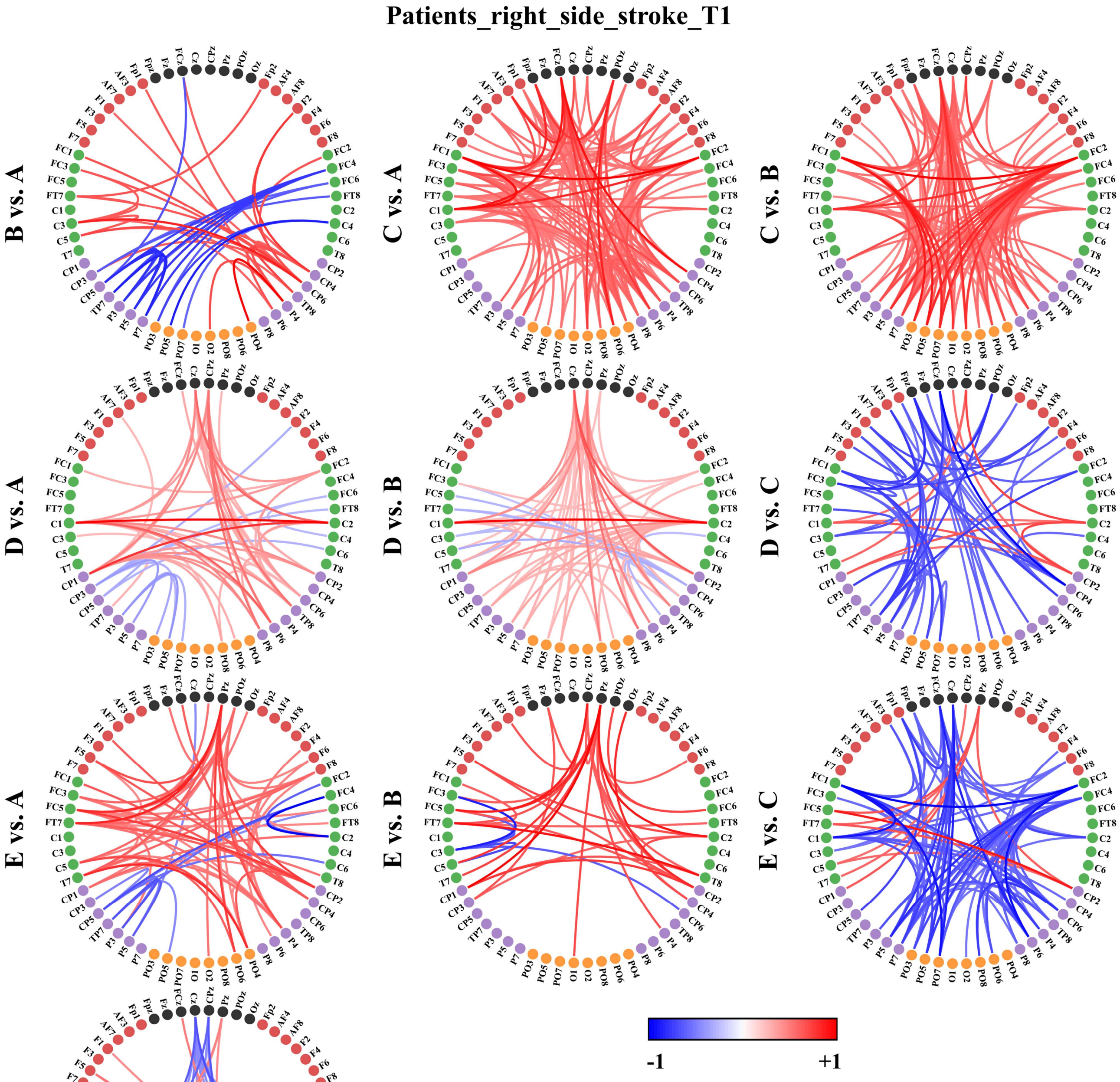

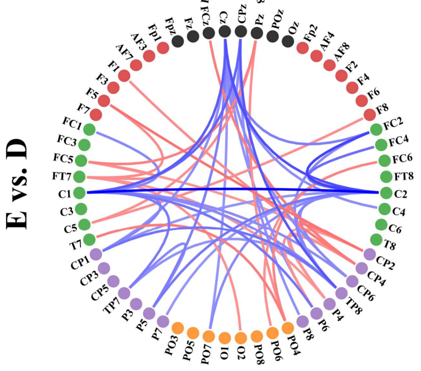

X vs. Y	B vs. A	C vs. A	C vs. B	D vs. A	D vs. B	D vs. C	E vs. A	E vs. B	E vs. C	E vs. D
X > Y	**0.001**	**0.001**	**0.001**	**0.001**	**0.001**	**0.032**	**0.001**	**0.001**	**0.022**	**0.002**
X < Y	**0.002**	None	None	**0.004**	**0.006**	**0.001**	**0.010**	**0.018**	**0.001**	**0.001**

Supplementary Figure 16. Results of microstate-wise functional connectivity comparisons for patients with right-sided stroke at T1. For X vs. Y, the red color indicates X > Y, while the blue color indicates the opposite pattern. Color depth indicates the size of the difference. Only the significant connected components are displayed. *p*-values are also provided in the figure.

Patients_T1 vs. Patients_T0 (microstate-wise FC)

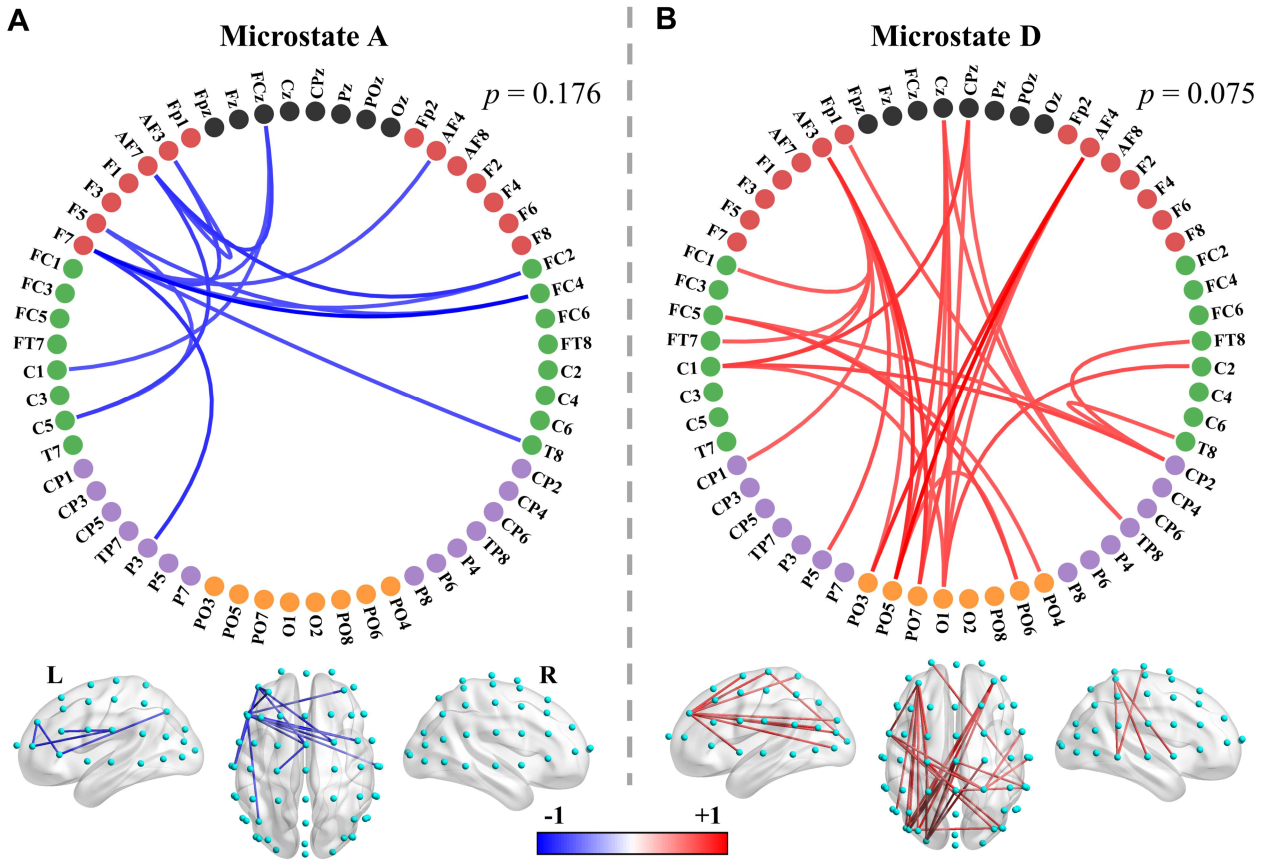

Supplementary Figure 17. Microsite-wise functional connectivity differences between patients at T0 and T1. (**A**) Patients at T0 versus Patients at T1 for functional connectivity of microstates A. (**B**) Patients at T0 versus Patients at T1 for functional connectivity of microstates D. For X versus Y, the red color indicates greater connectivity for X versus Y, and the blue color indicates greater connectivity for Y versus X. Color depth represents the size of the connection difference. For each comparison, the smallest *p*-value and corresponding connected component are provided.

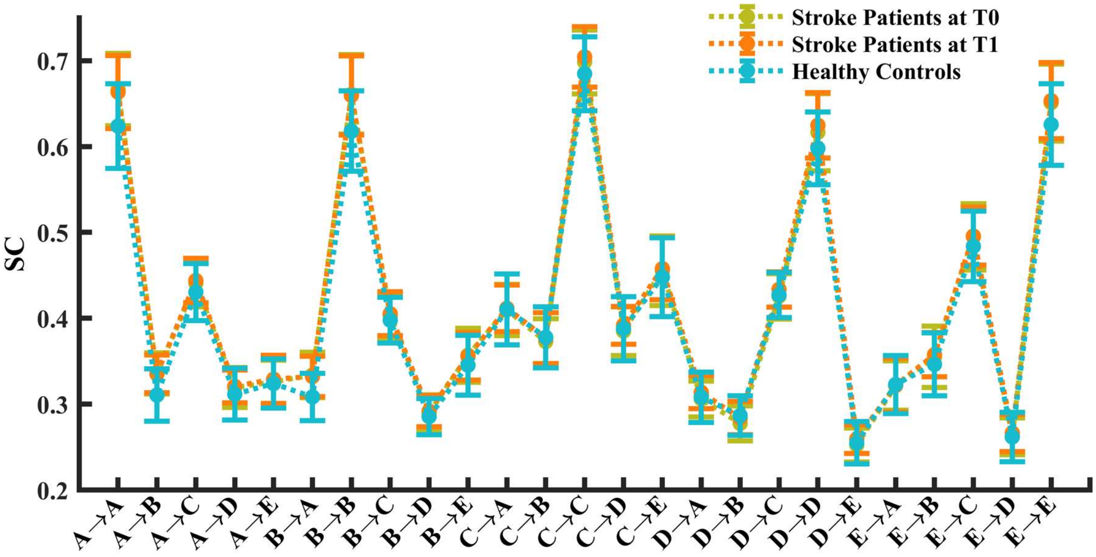

SC pairs	SC_{AA}	SC_{AB}	SC_{BA}	SC_{BB}	SC_{DD}	SC_{EE}
P_T0 vs. HC	0.001	0.002	0.001	0.002	NS	0.048
P_T1 vs. HC	0.003	0.001	0.001	0.002	0.018	0.032

Supplementary Figure 18. Results of comparisons between patients and healthy controls in SC. For each group, a dashed line links the mean across all SC pairs. The standard deviation is also displayed for each SC pair. *p*-values of SC pairs with significant differences between patients and controls are provided (without Bonferroni-Holm correction). NS: nonsignificant, P: patients, HC: healthy controls.

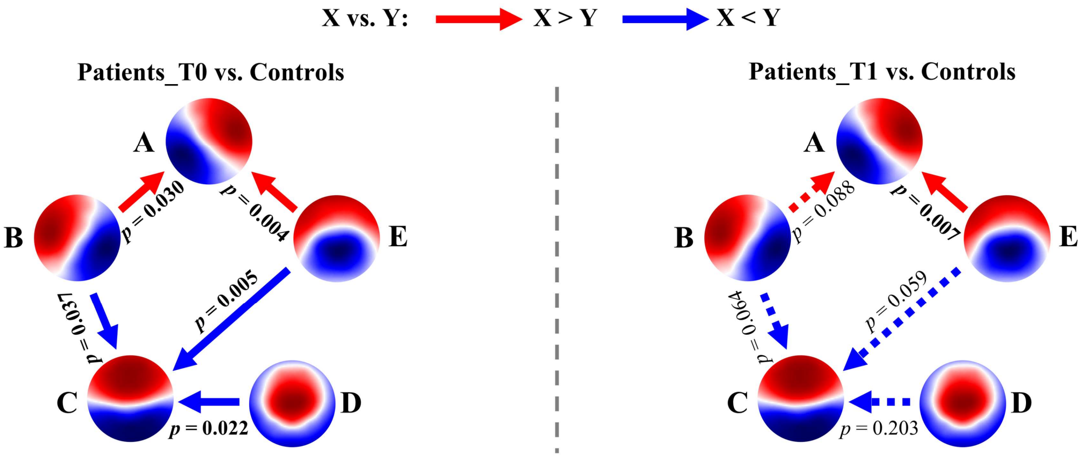

Supplementary Figure 19. Results of comparisons between patients and healthy controls in TP. The red color indicates increased transition probability between two microstate classes, and the blue color indicates decreased transition probability between two microstate classes. The solid arrows and bolded *p*-values indicate significant differences (without Bonferroni-Holm correction).

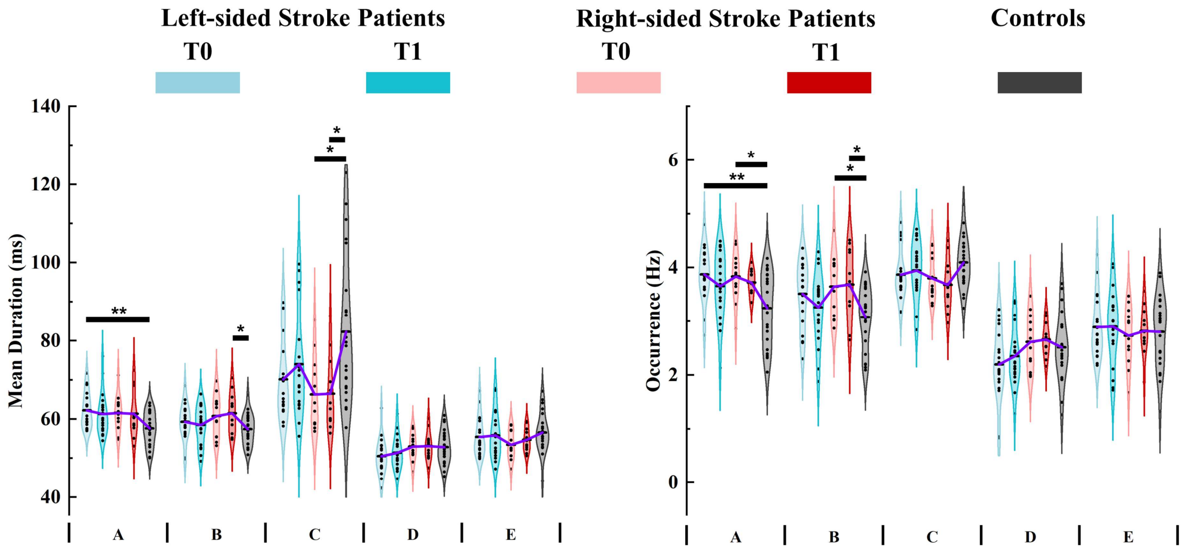

Supplementary Figure 20. Results of microstate parameter analysis for subgroups of patients and controls. Pairwise comparisons (Patients with left-sided stroke versus Patients with right-sided stroke at T0/T1; Patients with left-sided stroke at T0/T1 versus Controls; Patients with right-sided stroke at T0/T1 versus Controls) were performed for the mean duration, occurrence. The means of the five groups are linked by the solid purple line. *$p < 0.05$; **$p < 0.01$.

Correlation

Microstate-wise FC ⟷ FMAL

T0 T0

MS	A	B	C	D	E
NEG *p*	**0.016**	**0.024**	**0.045**	**0.043**	**0.023**

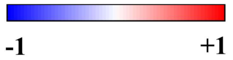

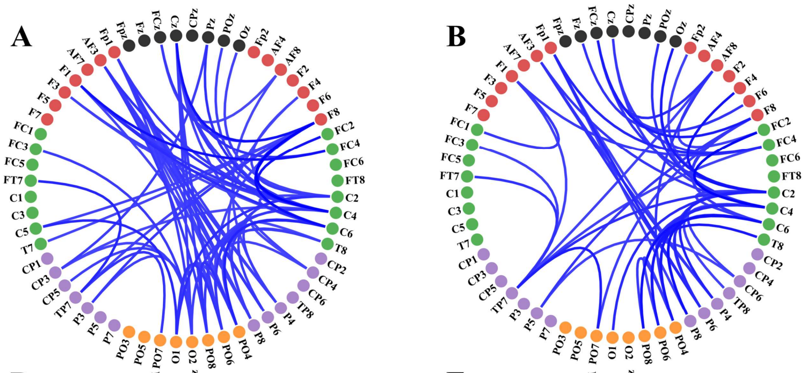

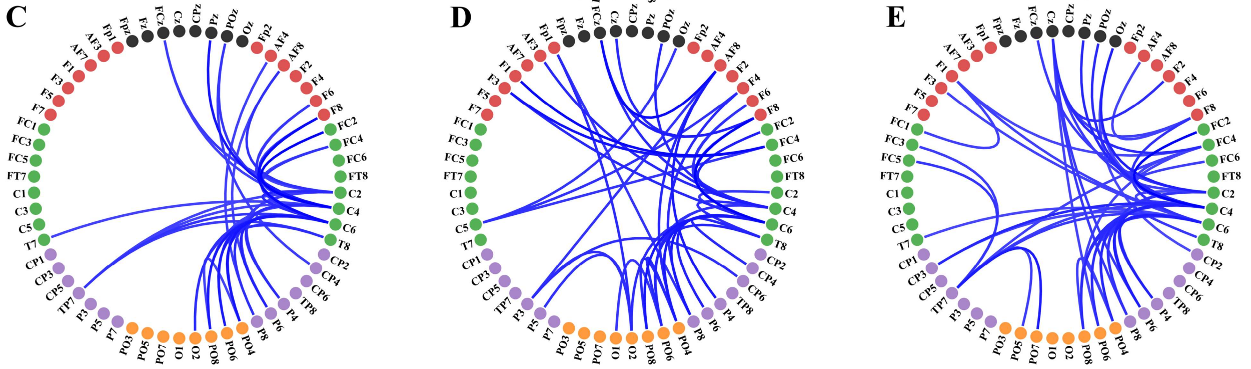

Correlation

Microstate-wise FC ⟷ FMAL

T1 T1

MS	A	B	C	D	E
NEG *p*	**0.021**	**0.030**	**0.020**	**0.028**	0.142

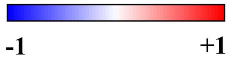

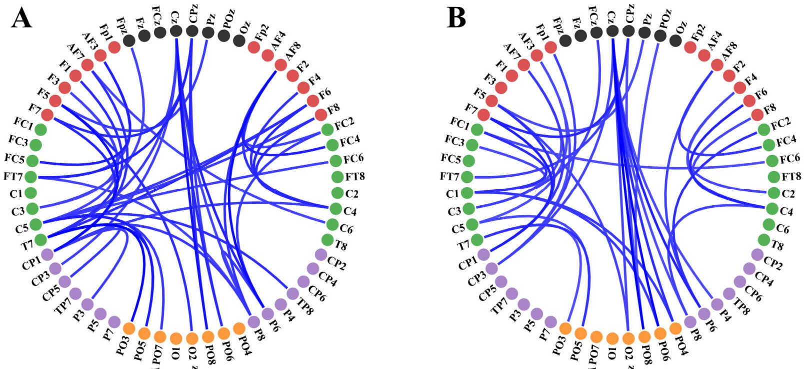

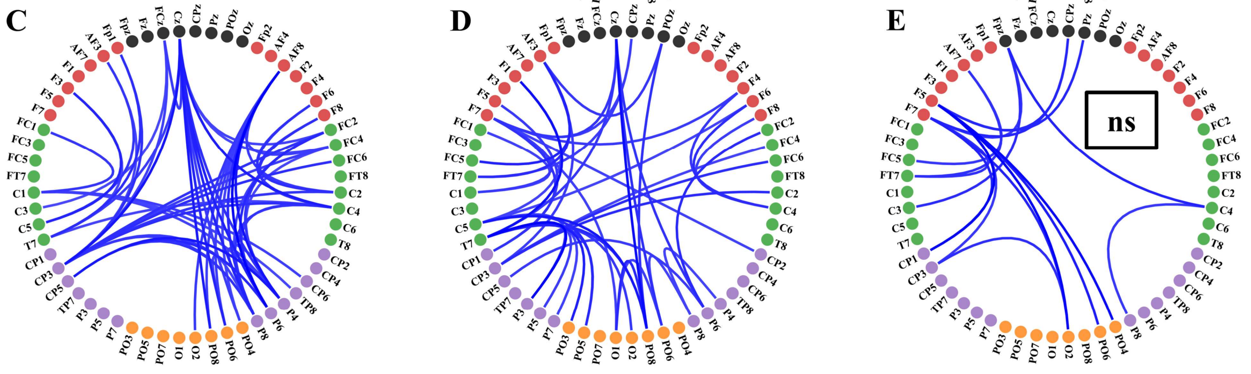

Supplementary Figure 21. Results of correlation analysis between microstate-wise functional connectivity and FMAL. For X vs. Y, the blue color indicates the negative correlations. Color depth indicates the size of the correlation. For each microstate class, the smallest *p*-value and corresponding connected component are provided. Only the connected components with $p < 0.200$ are displayed. MS: microstate class, NEG: negative, ns: nonsigificant.
